# Neuron–Glia Crosstalk in the Regulation of Astrocytic Antioxidative Mechanisms Following CNS Injury

**DOI:** 10.3390/antiox14121415

**Published:** 2025-11-27

**Authors:** Piotr K. Zakrzewski, Tomasz Boczek

**Affiliations:** Department of Molecular Neurochemistry, Faculty of Health Sciences, Medical University of Lodz, 92-215 Lodz, Poland

**Keywords:** astrocytes, antioxidative mechanisms, CNS injury

## Abstract

Astrocytes play a key role in maintaining redox balance and supporting neuronal survival within the central nervous system (CNS). Their antioxidant machinery, primarily involving the Nrf2–ARE (nuclear factor erythroid 2-related factor 2–antioxidant response element) pathway, glutathione (GSH) metabolism, and mitochondrial function, enables the removal of reactive oxygen and nitrogen species (ROS and RNS) and supports neuronal resistance to oxidative stress. Effective communication between neurons and astrocytes coordinates metabolic and antioxidative responses via glutamate-, nitric oxide-, and calcium-dependent signalling. Disruption of this crosstalk during traumatic injury, ischemia, or neurodegenerative disease causes redox imbalance, neuroinflammation, and excitotoxicity, which contribute to progressive neurodegeneration. Astrocytic Nrf2 activation reduces oxidative damage and inflammation, while its suppression encourages a neurotoxic glial phenotype. Current evidence emphasizes various therapeutic strategies targeting astrocytic redox mechanisms, including small-molecule Nrf2 activators, GSH precursors, mitochondria-targeted antioxidants (MTAs), and RNA- and gene-based approaches. These interventions boost the antioxidant ability of astrocytes, influence reactive cell phenotypes, and support neuronal recovery in preclinical models. Although there are still challenges in delivery and safety, restoring neuron–glia redox signalling offers a promising strategy for neuroprotective treatments aimed at reducing oxidative stress-related CNS injury and disease progression.

## 1. Introduction

The central nervous system (CNS) is remarkably susceptible to oxidative stress, a state resulting from an imbalance between the production of reactive oxygen and nitrogen species (ROS and RNS) and cellular antioxidative mechanisms. Several intrinsic features of the CNS make it particularly prone to oxidative damage: neurons consume large amounts of oxygen to sustain their high metabolic activity, contain abundant polyunsaturated lipids that can be easily oxidized, and the overall antioxidant capacity of the brain is relatively limited compared to other tissues. When ROS and RNS accumulate beyond physiological levels, they affect proteins, lipids, and nucleic acids, disrupting membrane integrity, enzyme function, and genetic stability. Consequently, oxidative stress has emerged as a central and unifying pathological mechanism contributing to a broad spectrum of neurological disorders, including ischemic stroke, traumatic brain injury (TBI), chronic neurodegenerative diseases, and inflammatory conditions. Although these diseases may differ in their origin and progression, they share several converging molecular pathways, most notably mitochondrial dysfunction, excitotoxicity, inflammation, and oxidative injury that lead to neuronal death and functional decline [1,2,3].

Mitochondria are both a major source and a target of oxidative damage, as an imbalance in the electron transport chain enhances ROS generation, further destabilizing mitochondrial integrity and energy metabolism. In neurons, glutamate excitotoxicity resulting from excessive stimulation of its receptors, elevates intracellular calcium and triggers activation of enzymes that produce free radicals, further contributing to progressive neurological deterioration [4,5]. Within this context, astrocytes play a fundamental and multifaceted role in maintaining redox balance and protecting neurons against oxidative injury. As the most abundant glial cell type, astrocytes provide essential metabolic and antioxidant support to neurons, regulate extracellular ion and neurotransmitter concentrations, and form part of the blood–brain barrier (BBB). Their antioxidant systems are particularly robust, including high concentrations of glutathione (GSH), superoxide dismutase (SOD), catalase (CAT), and GPx (GSH peroxidase) [6,7,8,9].

When astrocytic redox regulation fails, neurons become particularly vulnerable to oxidative damage. In cerebral ischemia, the sudden interruption of blood flow deprives neural tissue of oxygen and glucose, rapidly affecting ATP synthesis. The resulting energy undersupply disrupts ionic homeostasis, leading to excessive glutamate release. The consequent calcium influx triggers the activation of enzymes such as nitric oxide synthase, phospholipases, and proteases, all of which contribute to the formation of ROS and RNS. Upon reperfusion, the abrupt reintroduction of oxygen further exacerbates oxidative stress by increasing mitochondrial electron leakage and activating xanthine oxidase. This oxidative burst results in lipid peroxidation, mitochondrial collapse, and ultimately neuronal death via necrosis and apoptosis [10,11,12]. A similar redox-driven cascade occurs in TBI and spinal cord injuries (SCI). The initial mechanical insult produces immediate structural damage and vascular disruption, followed by a secondary phase characterized by mitochondrial dysfunction, calcium dysregulation, and massive oxidative and inflammatory activation. Microglia and infiltrating immune cells release ROS, RNS, and proinflammatory cytokines, amplifying tissue injury beyond the original lesion site. Persistent oxidative stress after trauma not only worsens acute neuronal loss but also promotes chronic neuroinflammation and delayed neurodegeneration, which are key contributors to long-term neurological deficits [13,14].

Oxidative stress is also a defining hallmark of chronic neurodegenerative disorders such as Alzheimer’s disease (AD), Parkinson’s disease (PD), and amyotrophic lateral sclerosis (ALS). In these conditions, prolonged mitochondrial dysfunction, impaired protein degradation, and accumulation of misfolded proteins lead to sustained ROS generation [5,15]. In PD, for instance, dopamine oxidation and mitochondrial complex I impairment produce free radicals that selectively damage dopaminergic neurons in the substantia nigra [16]. In AD, β-amyloid peptides catalyze ROS formation, disrupt mitochondrial function, and inhibit antioxidant enzymes, further promoting neuronal death [17]. Similarly, oxidative modification of lipids and proteins interferes with synaptic communication and signal transduction, perpetuating the degenerative process. Inflammation represents another powerful driver of oxidative stress within the CNS. Activated microglia and astrocytes produce ROS and RNS as part of the immune response to injury or infection. While transient activation can be neuroprotective, facilitating debris clearance and tissue repair, chronic activation leads to sustained oxidative and nitrosative stress [18,19]. This increases oxidative damage in neurons and further activates glial cells, creating a vicious cycle of inflammation and oxidative stress. In this complex network of interactions, neuron–glial communication emerges as a crucial factor in maintaining astrocytic antioxidative defence and overall redox homeostasis. Astrocytes continuously adjust their metabolic and antioxidant responses in accordance with neuronal activity and metabolic demands [8].

Metabolic interactions between neurons and astrocytes further contribute to the maintenance of redox homeostasis. When neuron–glial signalling is disrupted, the adaptive antioxidant responses of astrocytes are impaired, resulting in redox imbalance and increased neuronal vulnerability. In neurodegenerative diseases, loss of neuronal cues diminishes astrocytic metabolic coordination, reducing the capacity to neutralize ROS. Consequently, oxidative stress becomes self-sustaining, driving further neuronal death and disease progression [2,20].

Oxidative stress represents a central and shared pathogenic mechanism in virtually all forms of CNS injury. Astrocytes, through their metabolic adaptability and antioxidant capacity, serve as principal regulators of neuronal redox homeostasis. Their function, however, depends strongly on continuous communication with neurons.

Numerous reviews have summarized astrocytic antioxidant mechanisms. This review provides a conceptual synthesis that emphasizes the dynamic neuron–glia redox dialogue as a central integrative process shaping cellular responses after CNS injury. Unlike earlier accounts that mainly emphasized intracellular Nrf2–Keap1 (nuclear factor erythroid 2-related factor 2–Kelch-like ECH-related protein 1) signalling, we compile evidence from multiple cell types to propose a model of cross-cellular redox coordination linking astrocytic, neuronal, and microglial antioxidative processes networks. This review highlights how intercellular metabolic coupling and signal transduction together influence the effectiveness of redox modulation, providing a new systems-level view that integrates molecular, cellular, and potential translational elements of antioxidant neuroprotection. Understanding and enhancing this neuron–astrocyte crosstalk offers significant therapeutic potential by stimulating Nrf2 signalling, strengthening astrocyte metabolism, or restoring neuron–glial metabolic coupling, which could markedly improve the brain’s resilience to oxidative stress, particularly following CNS injury. Therefore, preserving the dynamic interplay between neurons and glial cells is not only fundamental for maintaining CNS homeostasis but also represents a promising direction for future neuroprotective interventions.

## 2. Astrocytic Antioxidant Machinery

The phase II antioxidant response includes detoxifying and antioxidant enzymes, whose expression is induced exclusively by de novo transcription and is governed by the transcription factor nuclear factor erythroid-2-related factor 2 (Nrf2, encoded by *NFE2L2*). The Nrf2 factor activates the antioxidant response via interaction with the ARE (antioxidant response element) of Nrf2-targeted genes. Given this, Nrf2 is regarded as a master regulator of antioxidant defence [21]. Nrf2 is a transcription factor sensitive to cellular redox status, forming heterodimers with MAF (musculoaponeurotic fibrosarcoma) family proteins, which allows them to recognize and bind to DNA regulatory motifs [8].

**Figure 1:**
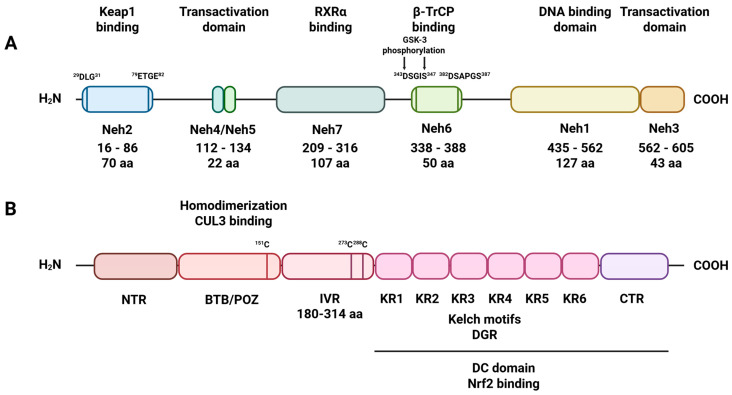
Schematic diagram of Nrf2 and Keap1 domains. (**A**) Nrf2 has seven primary domains, designated Neh1–Neh7. The Neh1 domain, part of the leucine zipper motif’s basic region, influences stability, DNA binding, and sMAF dimerization. Neh2 contains two interaction regions: the DLG motif (DLG) and the ETGE tetrapeptide motif (ETGE), which facilitate binding to Keap1. The Neh4, Neh5, and Neh3 domains are involved in Nrf2 transactivation. The serine-rich Neh6 domain controls Nrf2 stability; (**B**) Keap1 has three main domains. The BTB domain mediates Keap1 homodimerization and its interaction with CUL3. The IVR domain includes a critical cysteine residue connecting the BTB domain to the C-terminal Kelch/DGR domain. The Kelch/DGR domain binds Nrf2 via the Neh2 region.

The Nrf2 factor is composed of 605 amino acid residues, forming seven domains (Neh1–Neh7). The Neh1 domain (435–562 aa) contains a DNA-binding motif, which enables Nrf2 to interact with other transcription factors. Moreover, Neh1 stabilizes Nrf2 by binding to the ubiquitin-conjugating enzyme UbcM2. The main function of the Neh2 domain, located at the N-terminus of Nrf2, is interaction with Keap1. Neh3 (562–605 aa), 4, and 5 (112–134 aa) are involved in Nrf2 transactivation through interactions with coactivators. Specifically, Neh 3 interacts with the coactivator chromo-ATPase/helicase DNA-binding protein family member CHD6 (chromo-ATPase/helicase DNA-binding protein 6), whereas Neh 4 and Neh 5 interact with the CH3 domain of CBP (CREB-binding protein) [22]. Two motifs in Neh 6 (338–388 aa), DSGIS and DSAPGS, bind to the β-transducing repeat containing protein (β-TrCP). This protein acts as a substrate adaptor for the Skp1/CUL1/Rbx1Roc1 ubiquitin ligase complex. The DSGIS motif in Neh 6 is phosphorylated by GSK-3 (glycogen synthase kinase 3), which enhances β-TrCP to ubiquitin-dependent degradation of Nrf2. Additionally, the Neh 7 domain binds to retinoic X receptor alpha (RXRα) and suppresses the transcription of Nrf2 target genes [23,24] (Figure 1).

**Figure 2:**
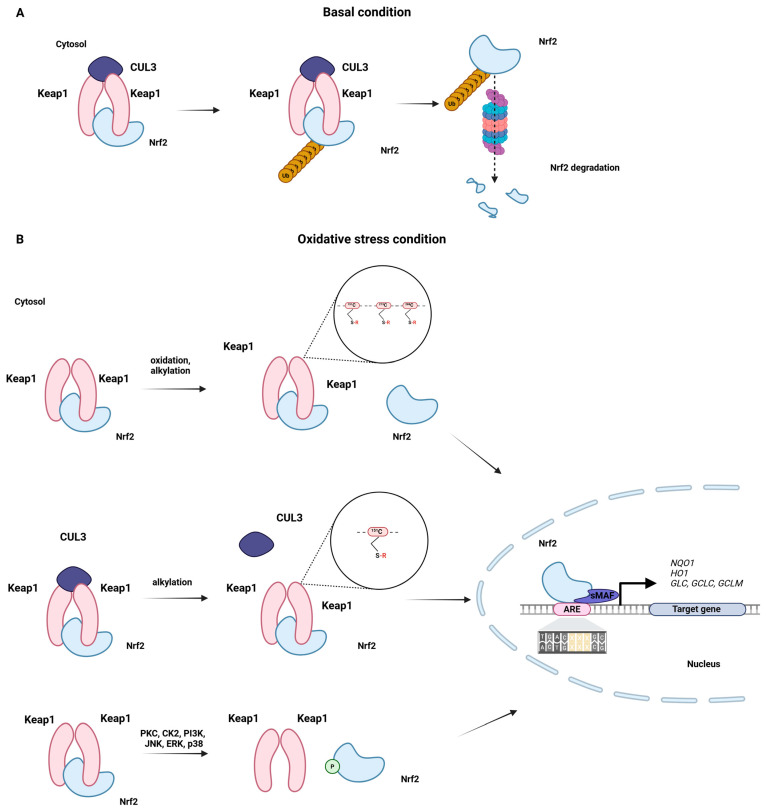
The schematic illustrates the Nrf2–Keap1 signalling pathway. (**A**) In basal conditions, Keap1 binds to the ETGE and DLG motifs on Nrf2, enabling Nrf2 to join the Keap1–Cul3 ubiquitin ligase complex, which tags Nrf2 for degradation via the proteasome. (**B**) Under oxidative stress conditions, an inducer (oxidation or alkylation) modifies a critical cysteine on Keap1, disrupting Keap1′s inhibitory complexes and preventing Nrf2 ubiquitination. This modification causes a conformational change in Keap1, releasing Nrf2 and preventing its ubiquitination, allowing it to escape degradation. Nrf2 then translocates into the nucleus, binds to the ARE, and activates genes encoding NQO1, HO-1, and GCL subunits C and M, boosting cellular defences against oxidative stress. As a result of oxidative stress, PKC, CSK2, PI3K, JNK, ERK, and p38 can induce phosphorylation-related translocation of Nrf2 to the nucleus.

When oxidative stress is not elevated, the Nrf2 factor is targeted for degradation by its endogenous inhibitor Keap1 via ubiquitin-mediated pathways [25,26]. Keap1 comprises 627 amino acid residues and belongs to the Kelch family, which contains a terminal BTB/POZ domain [27]. The Keap1 protein possesses within its structure five domains, i.e., the C-terminal region (CTR), double glycine repeats (DGR), tramtract and bric-a-brac (BTB) domain, the N-terminal region (NTR), and the intervening region (IVR), which are crucial for Keap1 molecular function [28] (Figure 1). The six repeated Kelch motifs (KR1-KR6) are located in the DGR domain and form a six-bladed propeller structure. The DGR and CTR domains together form the DC domain, which is necessary for Neh2 binding to Nrf2 [29]. The next structural element crucial for the antioxidative properties of Keap1 is the IVR domain (180–314 aa). The IVR localizes between the BTB and DGR domains and is rich in cysteine residues, which, under oxidative stress, undergo oxidation and alkylation. Specifically, the modification of cysteine 151, cysteine 273, and cysteine 288 alters the conformational state of the Keap1 protein, which, in effect, leads to the dissociation of Nrf2 from the Nrf2/Keap1 complex [30]. In turn, the liberated Nrf2 factor translocates from the cytosol to the nucleus, where, upon heterodimerization with small MAF (sMAF), it triggers the expression of antioxidative response genes [31]. The antioxidative response activated by Nrf2 requires the presence of the ARE element, with the consensus sequence 50-TGACxxxGC-30, found in the promoters of Nrf2 target genes [32] (Figure 2).

Nuclear translocation of Nrf2 occurs through the importin-alpha 5/importin-beta 1 import pathway [33]. Nrf2 has NESzip motif, the nuclear export signal co-localized with the leucine zipper (ZIP) domain. When Nrf2 combines with sMAFG through a ZIP-ZIP interaction, it enhances the retention of Nrf2 in the nucleus. The sMAFG-mediated detention of Nrf2 in the nucleus prevents its proteasomal degradation and stabilizes Nrf2 signalling [34]. Another mechanism supporting the inhibition of Keap1 involves the binding of Keap1 to the CUL3/RBX1 complex. Within the Keap1/CUL3RBX1/E3 ubiquitin ligase complex, Keap1 acts as a substrate adaptor, binding to RBX1 through the CUL3 scaffold. When the Nrf2 complex binds Keap1, it leads to the ubiquitination of Nrf2. Keap1 interacts with CUL3 via cysteine 151 in the BTB domain. Modifications such as alkylation or oxidation of cysteine 151 alter the structure of the BTB domain of Keap1. It disturbs the interac-tion between Keap1 and CUL3. This state results in Keap1 blockade, leading to the evasion of Nrf2 ubiquitination [35] (Figure 2).

Besides direct modification of cysteine residues in Keap1, the Nrf2 shuttling to the nucleus can be induced by the activation of kinases in response to the electrophilic or oxidative stimuli. Activated kinases phosphorylate Nrf2 at serine and threonine residues, which facilitates the dissociation of the Nrf2/Keap1 complex. Phosphorylation-related translocation of Nrf2 to the nucleus can be mediated by protein kinase C, casein kinase 2, phosphatidylinositol 3-kinase, PKR-like endoplasmic reticulum kinase, JNK (c-Jun N-terminal kinase), ERK (extracellular signal-regulated kinase), p38 MAPK (mitogen-activated protein kinase), and AMP-activated protein kinase. On the other hand, GSK-3 negatively regulates Nrf2 activity by phosphorylating different sites [36,37] (Figure 2). GSK-3 mediated Nrf2 phosphorylation directs Nrf2 to proteasomal degradation [38].

The Nrf2-ARE axis maintains antioxidative homeostasis in astrocytes by activating a plethora of antioxidant genes, encoding NAD(P)H quinone dehydrogenase (NQO1), heme oxygenase 1 (HO-1), and the two subunits of glutamate-cysteine ligase (GLC), GCLC (γ-glutamate-cysteine ligase catalytic subunit) and GCLM (γ-glutamate-cysteine ligase modifier subunit), engaged in GSH synthesis. Furthermore, Nrf2 also activates GPx, GSH S-transferases (GST), peroxiredoxins (Prx), thioredoxins (Trx), thioredoxin reductases (TrxR), and NADPH (nicotinamide adenine dinucleotide phosphate—reduced form) regenerating enzymes [39] (Figure 2). In addition to the aforementioned genes, Nrf2 upregulates *SQSTM1* (sequestosome 1), which encodes p62, a protein involved in autophagy [40].

Astrocytes play a key role in maintaining glutamate homeostasis, which, in turn, influences the balance of oxidative stress by regulating excitatory amino acids. They also help prevent excitotoxicity by releasing neurotrophic factors like glial-cell-line-derived neurotrophic factor (GDNF) and nerve growth factor (NGF), which promote neuronal survival [41,42]. During oxidative stress, astrocytes protect neurons by producing antioxidant compounds like GSH, ascorbate, and vitamin E, and by activating enzymes that neutralize ROS, including GST, GPx, TrxR, and CAT. This promotes better neuronal survival [43,44,45]. Neurons absorb GSH from the extracellular space directly or break it down with extracellular neuronal aminopeptidase N to form glycine and cysteine [46]. It has been confirmed that GSH-depleted astrocytes exhibit reduced neuronal protection against oxidative damage, as neurons lack sufficient substrates for GSH synthesis [47]. By boosting their ability to absorb cysteine, astrocytes enhance their capacity to synthesize GSH, which in turn strengthens their neuroprotective effect against oxidative stress [48].

Astrocytic defence against antioxidants also involves ascorbate recycling, which has the ability to remove ROS directly and also helps recycle oxidized vitamin E and GSH [49]. Recycled ascorbate is utilized within astrocytes or released into the extracellular space, where neurons can utilize it as part of their own antioxidant defence system. In neurons, ascorbic acid inhibits glucose consumption and stimulates lactate transport. Ascorbic acid modulates the astrocyte-neuron lactate shuttle; moreover, neurons synthesize glutamate, which stimulates astrocytes to release ascorbic acid in glutamatergic synaptic activity [50,51,52].

Astrocytes, due to their high expression of metallothioneins and ceruloplasmin, which are involved in metal binding and ion trafficking, also play a crucial role in sequestering metal ions, thereby preventing the generation of free radicals by redox-active metals [53,54].

## 3. Neuron–Astrocyte Crosstalk in Redox Regulation

Comparing Nrf2 expression between astrocytes and neurons reveals a precise pattern. Astrocytes are characterized by 100–1000-fold higher Nrf2 levels than neurons [55]. Nrf2-dependent antioxidative potential of astrocytes was confirmed in animal models, where Nrf2-deficient mice were more prone to oxidative stress than wild-type animals. Interestingly, Nrf2 ablation in cortical neurons did not alter their limited ability to protect against oxidative insults, rendering them unresponsive to Nrf2 activators [56,57]. However, Nrf2 expression appears to be necessary for the proper development of young neurons, which, like astrocytes, do not exhibit epigenetic inactivation of Nrf2. Mature neurons show significantly lower levels of Nrf2 promoter histone H3 acetylation compared to astrocytes. Repression of the *NFE2L2* gene occurs early in neuron development. In live animals at birth and in cells cultured for 2 days, Nrf2 expression and pathway activity are similar to those in astrocytes. However, by day 9 in culture, Nrf2 expression is repressed, and the promoter has less H3 acetylation [56]. An additional mechanism underlying neuronal unresponsiveness to oxidative stress is that neurons exhibit a greater capacity for Nrf2 degradation, dependent on CUL3 [55].

The weaker antioxidant defence in neurons can be attributed to the role of redox signalling in neuronal development [58,59]. The structural and electrophysiological development of neurons following ectopic expression of Nrf2 seems to result from the suppression of key developmental signalling pathways, including JNK and Wnt (wingless/integrated), whose activity is enhanced by redox signalling [60,61,62,63]. No such evidence is observed in astrocytes, where pronounced Nrf2 expression increases the antioxidative buffer capacity without affecting their development [64].

Limited Nrf2/ARE activity in neurons results in very low CAT and GSH expression levels. These antioxidative machinery components are strictly dependent on Nrf2 pathway activation. In both cases, Nrf2 regulates the expression of CAT and stimulates the transcription of key genes involved in GSH biosynthesis and regeneration [65,66]. Indeed, the expression of CAT and GCLC in cortical neurons is significantly lower than in astrocytes.

Neurons are highly metabolically active cells that place a high demand for ATP, which is necessary for maintaining their membrane resting potential [67]. During evolution, astrocytes have developed unique morphological and physiological features that support the proper functioning of neurons. They are able to take up substrates from the blood and metabolize them for local delivery to active synapses, thereby sustaining neuronal function. The main role of neurons in the CNS is neurotransmission, which is not only a highly energy-demanding process but also generates a large amount of reactive oxygen species (ROS), mainly associated with Ca^2+^ influx and glutamatergic stimulation [68,69,70]. Cross-talk between neurons and astrocytes is linked to glucose metabolism. Unlike astrocytes, neurons depend on the pentose phosphate pathway (PPP) for their glucose consumption. This pathway helps regenerate NADPH levels, which are necessary for effective reduction in GSH, the brain’s most abundant antioxidant [71,72,73].

Neurons also rely on GSH biosynthetic machinery to regenerate GSH, although in lower amounts, by utilizing amino acid precursors that result from the degradation of astrocytic GSH [74,75,76]. Astrocytes release GSH in response to oxidative stress stimuli. GSH and glutathione disulfide (GSSG) liberation from astrocytes involves the multidrug resistance protein 1 (Mrp1) transporter exclusively, but not Mrp5 (multidrug resistance protein 5) [77]. GSH precursors sequestered in the extracellular space are not only utilized to scavenge ROS, but also shuttle into nearby neurons [78,79].

**Figure 3:**
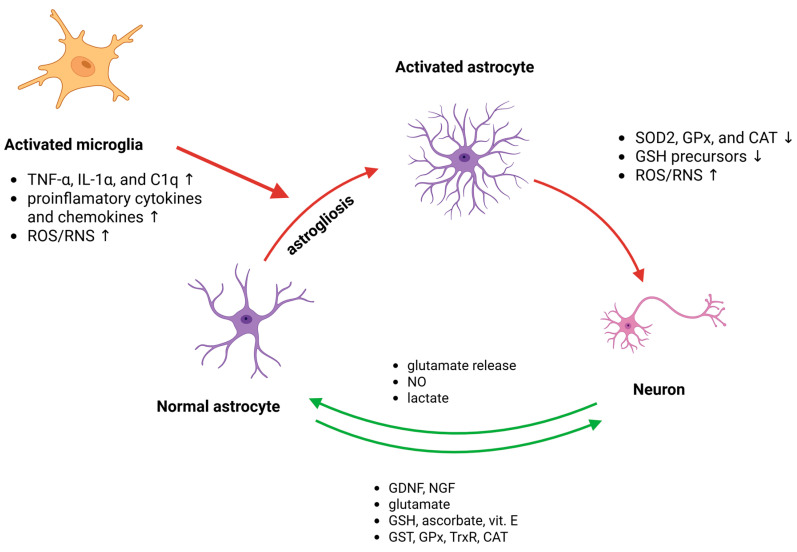
The primary molecular mechanisms involve responses and interactions among astrocytes, microglia, and neurons. Under physiological conditions (green arrows), astrocytes sustain homeostasis by releasing antioxidants, breaking down ROS/RNS, supplying energy, and absorbing and metabolizing neurotransmitters. In pathological states (red arrows), astrocytes can be stimulated by signals from activated microglia and activated astrocytes, leading to excessive release of free radicals and proinflammatory cytokines, glial scar formation, and disrupted regulation of excitatory amino acids, thereby worsening neurological injury.

Research also suggests that astrocytes may play a role in shielding neurons from ROS-induced injury by clearing damaged mitochondrial membranes. This mechanism potentially includes transmitophagy, a process by which functional mitochondria are transferred from astrocytes to neurons, where they provide the defence machinery for neurons [80]. The aforementioned phenomenon was described in the stroke model; however, to date, it remains questionable, as the observed damaged mitochondria might have originated from neuron-associated astrocytes rather than neurons [81,82]. On the other hand, it was confirmed that free fatty acids produced in the degradation of neuronal mitochondria can be transferred in ApoE+ (apolipoprotein E) lipid complexes to astrocytes, where the astrocyte mitochondrial β-oxidation pathway metabolizes them [83] (Figure 3).

## 4. Neuronal Signals That Boost Astrocytic Antioxidant Defences

Neuronal signals play a crucial role in boosting astrocytic antioxidant defences through multiple pathways [84,85]. Elevated neuronal activity leads to the secretion of glutamate and other soluble factors that activate the astrocytic Nrf2 pathway through group I metabotropic glutamate receptors and intracellular Ca*^2+^* signalling [86]. This creates a regulatory loop where astrocytic neuroprotective capacity matches the levels of adjacent synaptic activity. Additionally, NMDA receptor stimulation in astrocytes activates a phospholipase C-mediated pathway involving protein kinase Cδ (PKCδ) and cyclin-dependent kinase 5 (Cdk5), which phosphorylates Nrf2 and promotes its nuclear translocation, thereby inducing antioxidant gene expression [55]. Neurons also enhance their own antioxidant defences through synaptic NMDA receptor activity, which boosts the Trx-Prx system by inactivating the thioredoxin inhibitor (Txnip) and upregulating genes that reactivates Prx [87]. These mechanisms collectively ensure robust antioxidant protection against oxidative stress [20] (Figure 3).

Neurons are unresponsive to direct redox changes through Nrf2 activation. Instead, they rely on activity-mediated calcium-dependent pathways to increase the expression of Nrf2 target genes, even without Nrf2 activation. This phenomenon occurs through an alternative transcription factor, activator protein 1 (AP-1), which links synaptic activity to neuronal redox pathways. Interestingly, the AP-1 recognition site is embedded within ARE motifs [88,89]. When it comes to astrocytes, neuronal activity functions like a switch through mechanisms involving cAMP/PKA (protein kinase A)-dependent CREB (cAMP response element-binding protein) activation, increasing the production of primarily antioxidant molecules, such as GPx3 and SOD3, which are released outside the cells. GPx3 and SOD3 can be regarded as a part of an important mechanism of non-cell-autonomous astrocytic antioxidant support for neurons [85].

## 5. Astrocytic Response Pathways to Neuronal Signals

Redox-based communication between neurons and astrocytes is crucial for maintaining cellular homeostasis in the brain. Astrocytes provide essential antioxidant support to neurons through the Nrf2 pathway, which regulates a large cohort of antioxidant genes, while neurons have weak Nrf2 activity but compensate through activity-dependent antioxidant gene regulation [20]. This intercellular coupling involves both metabolic and functional connections mediated by gap junctions and hemichannels, allowing small molecule diffusion and neurotransmitter recycling [90]. The antioxidant and bioenergetic systems are tightly coupled between these cell types, with neurons metabolizing glucose through the PPP to maintain reduced GSH, dependent on energy substrate supply from astrocytes [76]. This neuron–astrocyte cross-talk ensures brain bioenergetic and redox homeostasis in health [91] (Figure 3).

Astrocytes respond to neuronal signals through multiple distinct pathways, enabling bidirectional communication in the brain. When neurons release neurotransmitters, astrocytes can detect these signals and respond with intracellular calcium elevations ([Ca^2+^]i) [92,93]. These calcium responses can remain localized to specific astrocytic processes or propagate as waves throughout the cell and to neighbouring astrocytes [93]. Astrocytes utilize glutamatergic and nitric oxide-mediated signalling pathways to sense neuronal activity [92]. In response, astrocytes can release glutamate in a calcium-dependent manner, which then signals back to neurons through NMDA receptors, creating a feedback loop [93,94]. This neuron–astrocyte communication can influence various brain functions, including synaptic transmission and vascular regulation through astrocytic contact with cerebral arterioles [93]. Additionally, astrocytes undergo reactive astrogliosis in pathological conditions, involving molecular and morphological changes that can have both beneficial and detrimental effects [95] (Figure 3).

## 6. Pathological Scenarios of Neuron–Glial Redox Signalling

Pathological scenarios of neuron–glial redox signalling involve complex interactions between oxidative stress and neuroinflammation that contribute to neurodegeneration. Under normal conditions, redox signalling provides essential cellular communication through transient free radicals and redox sensors in enzymes, receptors, and transcription factors [96,97]. However, when oxidative stress increases, the effective reduction potential of redox pairs diminishes, shifting cell signalling toward proinflammatory and proapoptotic pathways, creating a vicious cycle between oxidative stress and neuroinflammation [96]. This pathological redox signalling manifests itself during and after CNS insults, such as TBI, stroke, as well as contributes to major neurodegenerative disorders, including AD, PD, Huntington’s disease (HD), and ALS [97]. Cross-talk between proinflammatory and oxidative signals leads to neuronal damage via concurrent toxic pathways [98,99]. In prion diseases, altered redox balance facilitates protein misfolding and aggregation, inducing microglial activation and further redox stress [100]. CNS injuries, including traumatic insult, stroke, or neurodegenerative damage, are accompanied by cell death, inflammation, and oxidative stress.

### 6.1. Neuron–Glial Redox Signalling Alteration

Changes in neuron–glial redox signalling following central nervous system (CNS) injury involve complex interactions between oxidative stress and neuroinflammation, which critically shape neurological outcomes. Oxidative pressures alter molecular functions via redox-sensitive enzymes, receptors, and transcription factors, with thiol-based sensors being particularly vulnerable to oxidative modifications [96]. When oxidative stress rises, the reduction potential of redox pairs diminishes, shifting signalling toward proinflammatory and proapoptotic pathways and reinforcing a destructive cycle between oxidative stress and inflammation [96].

CNS injury initiates a graded neuroglial activation programme encompassing neurons, glia, and endothelia, which is evolutionarily conserved for protection and repair [101]. However, excessive cross-talk between oxidative and proinflammatory signals leads to neuronal damage and neurodegeneration [98]. Dysregulation of redox homeostasis, a fundamental aspect of CNS development, function, and ageing, contributes to pathological outcomes [99]. Pathological cascades following CNS trauma include oxidative stress, neuroinflammation, mitochondrial dysfunction, and neuronal apoptosis [102]. The release of cytokines such as TNF-α (tumour necrosis factor alpha), IL-1β, and IL-6 amplifies neuronal damage and impairs regeneration, whereas anti-inflammatory cytokines, including IL-10, IL-4, and IL-33, exert protective effects [103].

The Nrf2 pathway is a central regulator of antioxidant defence and inflammation control in acute CNS injury. In TBI and ischemic stroke, Nrf2 activation limits NF-κB (nuclear factor kappa B)–driven proinflammatory responses, reducing secretion of cytokines such as TNF-α, IL-1β, and IL-6 (interleukin 1β, 6) [104]. In SCI, Nrf2 activation counteracts ROS and cytokine production through NF-κB crosstalk [105]. Nrf2 induces the expression of antioxidant and detoxifying genes, including HO-1, NQO1, and enzymes that support GSH synthesis [106,107]. Astrocytes play a role in ischemic stroke, as they show cell-specific Nrf2 activity crucial for neuronal survival [108]. Nrf2 deficiency enhances NF-κB activation and delays motor/cognitive recovery after TBI [104,109,110]. Thus, Nrf2 signalling protects against oxidative damage and inflammation in both acute injuries and chronic neurodegeneration, making it a promising therapeutic target [56,111].

Another pathological mechanism occurring after CNS insult involves reactivation of microglia. Astrocyte activation occurs through complex molecular mechanisms involving reciprocal communication with microglia. Microglia are activated earlier than astrocytes and promote astrocytic activation, while activated astrocytes can both facilitate distant microglial activation and inhibit local microglial activities [112]. Activated microglia stimulate astrocytes via the secretion of TNF-α, IL-1α, and C1q (complement component 1q), which promote astrocytes toward the A1 phenotype. A1 astrocytes downregulate antioxidant enzymes, such as SOD2, GPx, and CAT, thereby diminishing their ability to supply neurons with GSH precursors and enhancing oxidative damage [113]. Additional molecules involved in astrocyte activation include vital substances such as ATP, regulatory hormones such as gonadal steroids, and injury-induced cytokines and chemokines [114]. This reciprocal interaction between microglia and astrocytes drives reactive gliosis, leading to glial scar formation that isolates damaged regions but could also impede axonal regeneration [115,116].

The third scenario leading to reduced antioxidative response in neuron–glial interactions involves excitotoxic glutamate and redox imbalance. Excitotoxic glutamate-redox imbalance represents a critical pathological mechanism following CNS injury, involving complex interactions between glutamate signalling and oxidative stress. Microglia contribute to excitotoxicity through the glutamate/cystine antiporter system xc^−^, which regulates both glutamate release and cellular redox balance, with high GSH:GSSG ratios predicting neurotoxic potential [117]. TBI disrupts normal glutamate and GABA homeostasis, creating excitation-inhibition imbalances that evolve through acute, subacute, and chronic phases [118]. The redox biology of excitotoxicity involves NMDA receptor modulation, the oxidative conversion of DOPA to the neurotoxic TOPA quinone, and the liberation of zinc from intracellular proteins, linking glutamate neurotoxicity to oxidative cellular cascades [119].

The consequence of CNS injury can also be the liberation of damage-associated molecular patterns (DAMPs) from insulted neurons [120]. Neuronal DAMPs play a crucial role in activating astrocyte-mediated responses that can exacerbate neurodegeneration. Chang et al. (2024) demonstrated that factors released from dying neurons signal through the receptor for advanced glycation end-products (RAGE) to activate astrocytic RIPK3 (receptor-interacting serine/threonine-protein kinase 3) signalling, promoting neuroinflammation and further dopaminergic cell death in PD models [121]. This creates a destructive cycle where neuronal death perpetuates additional neurodegeneration through inflammatory astrocyte activation. The oxidative stress component involves complex interactions between neurons and astrocytes. Reyes et al. (2012) demonstrated that NMDA receptor activation in neurons triggers the release of extracellular superoxide via NOX2 (NADPH oxidase 2), resulting in oxidative stress in neighbouring neurons and astrocytes [122]. However, astrocytes can also provide neuroprotection by regulating oxidative signalling in a controlled manner. Haskew-Layton et al. (2010) found that low-level hydrogen peroxide production in astrocytes protects neurons from oxidative stress through Nrf2-independent pathways, while higher levels become neurotoxic, highlighting the dual role of astrocytic oxidative responses [123].

In CNS injuries like stroke and TBI, the accumulation of iron leads to significant brain damage by promoting oxidative stress and triggering a form of cell death called ferroptosis. Ferroptosis, an iron-dependent programmed cell death, plays a crucial role in CNS injury pathogenesis through disrupted iron metabolism, GSH depletion, and lipid peroxidation [124]. Iron-mediated ROS generation in astrocytes represents a critical mechanism in neurodegeneration and oxidative stress. Astrocytes demonstrate sensitivity to acute iron overload, with iron entry impairing cellular reducing potential and promoting ROS production, ultimately leading to cell death through mitochondrial dysfunction [125]. Iron accumulation in cultured astrocytes occurs in a time- and concentration-dependent manner, causing transient increases in intracellular ROS and mild cytotoxicity. However, astrocytes possess adaptive mechanisms, including upregulation of ferritin and modulation of transferrin receptor levels to manage iron homeostasis [126]. Even iron oxide nanoparticles induce transient ROS production and ferritin upregulation, though astrocytes remain viable despite elevated iron content [127]. Notably, astrocytes exhibit greater resistance to oxidative stress compared to oligodendroglia, due to their higher GSH content and lower iron levels, making them less susceptible to ROS-mediated damage [128].

Astrocytes serve as key regulators of brain iron metabolism, efficiently accumulating iron ions and iron-containing compounds through specific transport proteins while storing iron in ferritin to protect against toxicity [129]. Under normal conditions, astrocytes have a strong capacity for iron transport and are positioned near blood vessels, making them key regulators of brain iron homeostasis despite having lower metabolic iron requirements than oligodendrocytes [130]. In neurodegenerative diseases, astrocytes become primary targets of iron neurotoxicity, leading to deficits in the glutamate/GABA-glutamine shuttle, the antioxidative machinery, and energy metabolism that facilitate neurodegeneration [131]. Iron accumulation occurs across diverse neurological conditions, including PD, AD, and SCI, due to dysregulated iron homeostasis mechanisms. Recent advances have highlighted ferroptosis as a key iron-dependent cell death pathway in the lipid-rich CNS environment [132]. Following ischemic stroke, glial cells demonstrate promising therapeutic targets due to their involvement in iron transfer between glia and neurons, indicating critical glia-neuron crosstalk in mediating ferroptosis-related pathology [133]. Ferroptosis affects multiple CNS cell types, including glial cells, neurons, and pericytes, with iron overload and lipid reactive oxygen species accumulation contributing to neuronal damage [134].

The NF-κB and JAK/STAT (Janus kinase/signal transducer and activator of transcription) signalling pathways play crucial roles in promoting neuroinflammation in astrocytes, particularly following SCI, when they initiate gene expression associated with astrogliosis and the production of proinflammatory factors. These pathways are interconnected, as NF-κB activation leads to the release of IL-6, which in turn activates STAT3 signalling [135]. The dominance of proinflammatory signalling over antioxidative responses involves complex molecular cross-talk between NF-κB and Nrf2 pathways [136]. Nrf2 and NF-κB are transcription factors that regulate antioxidant and inflammatory pathways in opposite ways [119]. In the absence of Nrf2, pronounced NF-κB activity leads to increased cytokine production [136].

Astrocytes play critical roles in neurological recovery following CNS injury by coupling their metabolism with that of neurons. Under normal conditions, astrocytes use glycolysis, while neurons rely on oxidative metabolism. Astrocytes provide metabolites, such as lactate and amino acid precursors, to support neuronal energy needs and maintain redox balance [137]. Following TBI, this metabolic cooperation is disrupted, with glucose oxidative metabolism being more severely impaired in neurons than in astrocytes [138]. Astrocytes respond to TBI with reactive astrogliosis, characterized by changes in gene expression, morphology, and function that can either promote neural repair or contribute to secondary injury [139]. Key astrocyte functions that affect neuronal survival include glutamate uptake, free radical scavenging, and cytokine production. Their contributions to long-term recovery involve the release of trophic factors, such as NGF, bFGF (basic fibroblast growth factor), TGFβ (transforming growth factor beta), PDGF (platelet-derived growth factor), BDNF (brain-derived neurotrophic factor), and CNTF (ciliary neurotrophic factor), as well as support for synaptic reorganization [140].

Pathological neuron–glial interaction following CNS injury weakens astrocytes’ antioxidant defences by inhibiting the Nrf2 pathway. This promotes neurotoxic cell behaviours, disrupts glutamate and metabolic interactions, impairs mitochondrial function, and leads to ongoing inflammation. This creates a feed-forward cycle involving oxidative stress, neurodegeneration, and glial dysfunction.

### 6.2. Neuron–Glia Redox Signalling in CNS Injuries

TBI is accompanied by increased inflammation and the generation of highly oxidative conditions, which are responsible for the so-called secondary strike effect [141,142,143]. The massive generation of ROS after injury disrupts astrocyte function and affects neuron–glia interactions. In the brain biopsies of brain injury patients, a reduced expression of astrocytic glutamate transporters (EAATs) has been found. This observation implicates a diminished ability of astrocytes to take up excitatory amino acids [144]. The accumulation of excitatory amino acids in the microenvironment can lead to mitochondrial calcium overload, intensifying oxidative stress and neuronal injury. The antioxidative potential and reduced generation of ROS after mechanical injury can be restored by an increase in hydrogen sulphide-containing (H_2_S) molecules, thus reducing the secondary strike effect. This protective outcome is a result of glutamate transporter (GLT-1) expression stimulation [145]. Mechanical stresses associated with brain injury may trigger a mitochondrial malfunction cascade in astrocytes, which then spreads to neurons [146].

Stroke is one of the main causes of disability or death worldwide. It is a clinical condition characterized by insufficient blood flow in the CNS. It can arise as a result of both hemorrhage or ischemia [147]. During and after the stroke incident, cell damage occurs mainly due to the elevated oxidative stress. Astrocytes, as key players in the antioxidative response in the CNS, play a dualistic role during stroke and recovery. They can be engaged in both neuroprotection and neurotoxicity, depending on their response to the microenvironmental conditions induced by stroke. In forebrain ischemia, an overexpression of astrocytic-specific SOD2 protects neurons from ischemia-related damage [148]. Furthermore, in cerebral ischemia, astrocytes are able to directly or indirectly transfer their functional mitochondria to neurons. Blockade of this exchange can effectively diminish neuronal damage resulting from cerebral ischaemia [81]. Stroke-induced elevation of oxidative stress stimulates astrocyte activation, which, in turn, contributes to impaired neurological recovery by forming glial scars [149,150]. Oxygen–glucose deprivation associated with stroke influences the expression, distribution, and activity of glial glutamate transporters. This results in impaired cellular glutamate uptake and reduced intracellular GSH levels, as confirmed in vitro in differentiated astrocytes [151].

### 6.3. Neurodegenerative-Related Neuron–Glial Redox Signalling

According to the amyloid cascade hypothesis of AD, the formation of amyloid β (Aβ) is linked to an increased vulnerability of brain tissue to oxidative signals [152]. Aβ secreted into the extracellular space tends to oligomerize, which, in turn, impairs NMDA receptor activity, leading to the generation of extracellular ROS and excessive calcium influx into neurons, eventually causing mitochondrial dysfunction [153,154,155]. Furthermore, Aβ aggregates into fibres and senile plaques, whose formation increases the oxidative stress and promotes apoptosis [156]. Post-mortem analysis of the brains of AD patients revealed that Nrf2 is predominantly expressed in the cytoplasm in hippocampal neurons, as well as it does not colocalize with beta amyloid plaques or neurofibrillary tangles. Moreover, the expression of Nrf2 in the nucleus is significantly decreased. These results indicate that Nrf2-driven transcription does not get activated in neurons during AD, even though oxidative stress is present [157]. On the other hand, recent studies analyzing post-mortem AD brain tissue have shown increased levels of Nrf2 and p62 in cells with high amyloid precursor protein or neurofibrillary tangles. Additionally, other Nrf2 targets and their gene transcripts are also elevated [40,158,159]. Regarding astrocytic Nrf2 expression in AD, Nrf2 seems to be expressed in the nucleus of some hippocampal astrocytes [157]. The evaluation of Nrf2-targeted genes in astrocytes has shown elevated NQO1 levels in hippocampal and frontal cortex neurons in AD brains [160,161,162]. A marked rise in NQO1 levels is observed in astrocytes surrounding plaques in both regions [161,162]. HO1 levels are elevated in neurons and astrocytes within the temporal cortex and hippocampus [163,164,165], and in microglia in the hippocampus of brains affected by AD. HO1 is also observed in astrocytes and microglia responding to mutated tau expression in the hippocampus of mice [158].

In various human pathologies, significant alterations in astrocytic function and morphology are observed. In the literature, the aforementioned changes are referred to as reactive astrogliosis. Processes that induce reactive astrocytes encompass inflammatory signals, loss of neuronal contact, and the direct consequences of disease-associated proteinopathy [113,166,167]. The abundance of reactive astrocytes is found in AD brain tissue, specifically Aβ or tau-rich areas [168,169,170]. Additionally, astrocytes may be stimulated by microglia activation to biosynthesize inflammatory factors, NO, and ROS, collectively agents that, in effect, promote a redox status imbalance [171]. In AD patients’ brains, astrocytic GSH secretion and GSH-synthesizing enzymes, such as GCL, are also dysregulated. At the early stages of AD, monomers of Aβ (mAβ) trigger GSH secretion, thus increasing astrocyte-related antioxidant potential. As Aβ aggregates increase in the form of oligomers, fibrils, or amyloid plaques, the astrocytic machinery that supports GSH release, especially via ABCC1, becomes impaired. This reduces the neural environment’s antioxidant buffering capacity. In effect, extracellular GSH release declines, possibly contributing to increased oxidative damage as the disease progresses [172].

PD is ranked as the second most common neurodegenerative disease after AD, and the leading cause of movement-related disorders worldwide [173]. The pathogenesis of PD is linked to α-synuclein (α-Syn) deposits in neurons. In the PD model, it was confirmed that α-Syn could be transferred from neurons to astrocytes, where it induces a strong response leading to astrocyte and microglia activation [174,175,176,177]. Furthermore, α-Syn stimulates astrocytes to excessive ROS generation and proinflammatory cytokines via TLR4 receptors, as it was demonstrated in an in vitro model [178]. Additionally, the accumulation of α-Syn may worsen oxidative stress in astrocyte-neuron co-culture systems, leading to lipid peroxidation and neuronal death [179,180].

The next neurodegenerative disease developing as a result of redox imbalance is ALS. In its familial form, app. 90% of patients are carriers of the SOD1 mutation [181]. Studies using mutant SOD1 in murine models and in vitro culture systems suggest that astrocytes play a role in propagating motoneuron injury and disease progression [182,183]. Astrocytes overexpressing the mutated SOD1 gene secreted elevated levels of TGFβ and inflammasome NLRP3. They also demonstrated an increased activation of the NF-κB pathway, thus leading to exacerbation of the inflammatory response in the model system [181].

## 7. Therapeutic Potential

Astrocytes, a major element in maintaining redox homeostasis in the brain, have become a greater focus as potential targets for intervention following CNS injuries. Possible scenarios of interventions targeting astrocyte redox imbalance include different approaches, such as the utility of small-molecule Nrf2 activators, replenishing of GSH/thiol pools, MTAs (mitochondria-targeted antioxidants) mimetics, modulation of astrocyte reactive phenotype, delivery of antioxidant enzymes/catalytic scavengers via biologics/nanodelivery, inhibition of astrocyte-related ROS sources, extracellular vesicles therapy to deliver antioxidant cargo, or RNA/gene interventions.

The first strategy involves activation of astrocytic Nrf2. Astrocytic Nrf2 activation strongly increases local antioxidant capacity and protects nearby neurons. Therefore, using small-molecule activators can be regarded as a potential strategy for Nrf2-related antioxidative responses in astrocytes. To date, a wide variety of molecules have been identified as Nrf2 activators, among which some are of potential clinical interest, as they can effectively stimulate an antioxidative programme via the Nrf2-ARE pathway.

Tert-butylhydroquinone (tBHQ) and sulforaphane were among the first compounds tested for potential application in minimizing oxidative stress. Research demonstrates that tBHQ and sulforaphane exhibit significant therapeutic potential in addressing redox imbalances in astrocytes. These two compounds function as an activator of the nuclear factor erythroid 2-related factor 2 (Nrf2) pathway, which preferentially occurs in astrocytes and provides neuroprotection [184]. Following TBI, tBHQ treatment effectively reduces astrocyte overactivation while enhancing Nrf2 nuclear accumulation and upregulating downstream antioxidative genes encoding HO-1 and NADPH-quinone oxidoreductase-1 [185]. The compound significantly attenuates oxidative stress markers, reduces malondialdehyde levels, increases superoxide dismutase activity, and decreases brain edema in mice after TBI [186]. Additionally, tBHQ demonstrates anti-inflammatory properties by reducing NF-κB activation and decreasing proinflammatory cytokines, including TNF-α, IL-1β, and IL-6 [187]. These findings collectively support tBHQ and sulforaphane as promising therapeutic agents for managing astrocyte-mediated redox imbalance in CNS injuries. However, in the case of tBHQ, a commonly used food preservative, it prevents the oxidation of unsaturated fats. According to some reports, the long-term exposure to TBHQ at higher doses (0.7 mg/kg) results in a series of side effects, including cytotoxic, genotoxic, carcinogenic, and mutagenic effects [188]. Similarly to tBHQ, sulforaphane activates the Nrf2 pathway, thus diminishing the risk of oxidative stress in an in vitro model of ischemia/reperfusion, when applied both as pre- and post-treatment after the injury [189,190].

Despite promising early results from preclinical studies, several challenges limit the practical use of astrocyte-focused antioxidative treatments. A major obstacle is crossing BBB, which restricts the availability of many small molecules. For example, the ability of tBHQ and sulforaphane to penetrate the BBB varies significantly. Sulforaphane shows measurable brain entry in mice, accumulating quickly in the ventral midbrain and striatum within 15 min after systemic administration, then reaching basal levels within 2 h, as evaluated by HPLC [191]. This brief but detectable presence coincides with upregulation of Nrf2-dependent genes in brain microvessels and tissue, indicating it can act directly within the CNS while maintaining BBB integrity. Conversely, evidence for tBHQ crossing the BBB is limited and mostly indirect [192]. tBHQ has shown neuroprotective effects in models of subarachnoid hemorrhage and TBI, both involving BBB compromise, but there is no quantitative pharmacokinetic data confirming its brain entry under normal conditions. Some studies also suggest that pre-ischemic exposure to tBHQ can worsen BBB disruption and oxidative stress in endothelial cells, suggesting that vascular effects depend on the context. Overall, these findings imply that while sulforaphane likely reaches effective CNS concentrations, tBHQ’s central activity may depend on the BBB damage or secondary systemic mechanisms [193]. A better understanding of their transport, distribution between the brain and plasma, and long-term vascular effects is essential before either can be safely developed as a therapeutic Nrf2 activators [194,195].

Omaveloxolone, a novel Nrf2 activator, shows promise as a therapeutic intervention following CNS injury through multiple mechanisms. In intracerebral hemorrhage models, omaveloxolone promoted Nrf2 nuclear accumulation, enhanced the expression of antioxidant enzymes, such as HO-1 and NQO1, and modulated microglial polarization from proinflammatory M1 to anti-inflammatory M2 phenotypes, while improving sensorimotor function [196]. The compound also demonstrated efficacy in Friedreich’s ataxia models by restoring mitochondrial Complex I activity, reducing oxidative stress markers, including lipid peroxidation and mitochondrial ROS, and protecting against mitochondrial depolarization [197].

Omaveloxolone demonstrates the ability to cross BBB and achieve measurable brain concentrations in preclinical studies. Specifically, in monkey studies, oral administration of omaveloxolone resulted in measurable, dose-dependent concentrations in brain tissue and in the lungs and liver. Omeveloxolone showed dose-linear plasma pharmacokinetics and dose-dependent induction of Nrf2 target genes, including in brain tissue, when tested as a potential drug for treating neurological conditions such as Friedreich’s ataxia [198]. However, the utility of omeveloxolone, as a novel drug targeting oxidative stress after CNS injury, demands further elucidation.

Ginsenosides, the primary bioactive components of ginseng, are another interesting group of compounds [199]. Among all the ginsenosides naturally present in ginseng, the administration of ginsenoside Rh1 (protopanaxatriol) can effectively inhibit the generation of hydrogen peroxide-related ROS, thus increasing the cell viability of rat primary astrocytes. Mechanistic studies indicated that Rh1 treatment recruited Nrf2 and c-Jun to ARE sequences and, in turn, stimulated the expression of phase II antioxidant enzymes, including HO-1, NQO1, SOD2, and CAT [200]. The antioxidative effect is also observed with ginsenoside Rg1, which protects against ischemic/reperfusion-induced neuronal injury via the miR-144/Nrf2/ARE pathway. miR-144 downregulates Nrf2 expression by targeting its 3′-untranslated region in PC12 cells cultured under oxygen–glucose deprivation conditions. Furthermore, administering Rg1 (20 mg/kg) to tMCAO rats significantly reduced ischemic injury by activating the Nrf2/ARE pathway [201]. As demonstrated in the in vitro model, the combination of ginsenoside Rb1, ginsenoside Rg1, schizandrin, and DT-13 (saponin compound from *Liriope muscari*), compounds derived from the Chinese traditional medicine formula ShengMai preparations, regulates the Nrf2/HO-1 pathway in H_2_O_2_-treated PC12 cells [202].

Ginsenosides have very limited ability to cross the BBB, with most research showing poor transport. Specifically, only Rg1 was detected in brain tissue after oral intake, and even then, only in trace amounts [203]. Further studies confirmed this, noting that Rg1′s passage through the BBB is restricted and usually undetectable in both normal and ischemic rat models [204]. Additionally, ginsenosides undergo extensive metabolic changes in the gastrointestinal tract and liver, which likely further reduce their BBB permeability [205]. While ginsenosides have promising pharmacological potential, their direct access to the brain remains highly limited.

Another potential therapeutic approach is the direct metabolic support to replenish the GSH pool in the CNS, particularly in astrocytes. Administration of cysteine precursors, such as N-acetylcysteine (NAC), can help maintain reduced GSH levels [206,207]. Regarding TBI, various preclinical studies in rats have demonstrated that NAC treatment reduces markers of oxidative stress, mitigates blast-related alterations in BBB integrity, and improves cognitive function after controlled cortical impact or blast injury [208,209,210]. NAC treatment following TBI stimulates the antioxidative programme via the Nrf2-ARE pathway [211]. The proof-of-concept pilot study “Pro-NAC” (ClinicalTrials.gov NCT01322009), which administered a combination of probenecid and NAC to children with severe TBI, has shown promising results that could be adopted in the future to develop a pharmacological strategy for TBI treatment. Probenecid, used as an adjuvant in this study, is a well-known inhibitor of ATP-binding cassette and solute carrier transporters, which prevents the transport of organic acids, such as NAC [212]. Further, a double-blind, randomized controlled study involving 81 patients showed that NAC supplementation benefits individuals exposed to blast-induced mild TBI. The study highlighted improvements in neuropsychological tests, reduced TBI symptoms, and faster recovery times compared with placebo.

Pharmacokinetic data show that oral administration of NAC has very poor bioavailability, ranging from 4% to 9% [213]. Although research indicates that NAC can cross the BBB [214]. When given orally at doses up to 70 mg/kg, NAC achieves cerebrospinal fluid (CSF) concentrations of approximately 9.26 ± 1.62 μM, with no significant adverse effects, and is well tolerated by individuals [215].

Administration of GSH appears to be the simplest strategy for enhancing antioxidant potential in the central nervous system following injury. However, GSH delivery is challenging due to its low bioavailability and poor stability, particularly in brain tissue. Novel delivery methods using nanomaterials and liposomes show promise for improving therapeutic efficacy [216,217]. More comprehensive approaches include enhancing cysteine uptake by upregulating the xc^−^ system (cystine/glutamate antiporter), increasing GSH synthesis via transcriptional factors such as Nrf2 and ATF4 (activating transcription factor 4 [218]. The upregulation of the xc^−^ system in astrocytes represents a promising but complex potential therapeutic approach for CNS injury, with evidence suggesting both protective and potentially harmful mechanisms. The xc^−^ system transporter critically mediates cystine/glutamate exchange, which is essential for GSH production and oxidative protection [219]. IL-1β has been reported to upregulate system xc^−^ activity in primary mouse astrocytes, increasing cystine uptake [220,221,222].

Mitochondria-targeted antioxidants (MTAs) show significant therapeutic potential for CNS injuries by addressing oxidative stress and mitochondrial dysfunction in both neurons and astrocytes. MTAs are synthetically modified antioxidant molecules designed to accumulate specifically in mitochondria. Various studies using mouse models of TBI or SCI have reported the efficacy of MTAs, such as Mito-Q (mitoquinone—ubiquinone derivative), SS-31 (elamipretide), and sinomenine, in enhancing neuroprotection by modulating astrocytic antioxidative potential. These antioxidants effectively prevent cardiolipin oxidation, reduce neuronal death, and decrease behavioural deficits and cortical lesion volume [223,224,225,226,227,228].

The literature data indicate that SS-31 and Mito-Q can effectively cross the BBB and target mitochondria. However, Mito-Q coaccumulation in mitochondria requires its conjugation to a lipophilic triphenylphosphonium cation [229,230,231,232]. In a mouse model of TBI, intraperitoneal administration of Mito-Q results in several-hundredfold accumulation within mitochondria, thereby exerting neuroprotective effects [228]. Sinomenine also passes the BBB when administered orally or intraperitoneally [233,234]. Oral dosing of sinomenine resulted in 80% bioavailability and tissue penetration, including the brain, within 45 min of administration, suggesting an advantage in clinical use [233]. Furthermore, the delivery of sinomenine to astrocytes can be effectively enhanced by conjugation to hydroxyl-terminated generation-4 PAMAM dendrimers, as demonstrated in a pediatric TBI rabbit model [235]. In ALS, mitochondrial dysfunction in SOD1G93A astrocytes promotes motor neuron degeneration through increased superoxide production and respiratory defects. MTAs, such as Mito-Q and Mito-CP (mito-carboxy proxyl), at nanomolar concentrations effectively prevent this dysfunction and restore motor neuron survival [236]. In the rat model of AD, the prolonged administration of another MTA, SkQ1 (plastoquinone-derivative antioxidant), has demonstrated a promising modality for the prevention and treatment of AD, characterized by a normalized gene expression profile of astrocytes and microglia, and a shift in the resting/activated microglia ratio toward a decrease in activated-cell density [237]. Furthermore, SkQ1 suppressed p38 MAPK signalling pathways and showed potential to prevent or slow disease progression [238]. SkQ1 shows promising evidence of BBB penetration and neuroprotective effects in CNS injury models, though direct pharmacokinetic studies are limited. In the TBI rat model, a single intravenous injection of SkQ1 (250 nmol/kg) demonstrated significant neurological benefits, reducing motor function impairment and improving neuronal survival [239]. The neuroprotective potential of MTAs can also be exploited in HD. Interestingly, as presented, the synthetic antioxidant XJB-5-131, when administered to HdhQ150 animals with well-established HD, improved weight gain, prevented neuronal death, reduced neuronal oxidative damage, and suppressed the decline in motor performance. Histological evaluation revealed, moreover, no significant differences in gliosis when compared to control animals [240].

The most advanced interventions include astrocyte reprogramming using mRNA (messenger RNA), siRNA (small interfering RNA), and miRNA (microRNA). These strategies show promising therapeutic potential for modifying redox imbalance following CNS injury [241,242,243]. Although the evidence remains largely preclinical. As demonstrated in a TBI mouse model, efficient delivery of BDNF mRNA loaded to a novel lipid nanoparticle (DA6LNP), which internalizes to the astrocytes, resulted in overexpression of BDNF in the brain, after just two consecutive intravenous injections. Animals administered with BDNF mRNA demonstrated a significant improvement in cognitive abilities compared with the control group [192]. Specifically, BDNF has been shown to reduce reactive oxygen species and increase antioxidant defences in astrocytes by activating Nrf2 [244,245]. Another approach to modulating astrocyte function following CNS injury involves siRNA-based strategies. Specifically, siRNA has been shown to effectively target and downregulate genes associated with astrocyte reactivity. Smith et al. (2018) [246] have reported a proof-of-concept use of the packaging RNA (pRNA)-derived three-way junction (3WJ) motif as a platform for delivering siRNAs to downregulate reactivity-associated genes. In that study, injecting anti-Lcn2-3WJs directly into the lesion in mice with SCI successfully reduced Lcn2 levels at both the mRNA and protein levels in vivo, thereby diminishing astrogliosis [246].

A promising platform to modulate the antioxidative potential in astrocytes is to use an astrocyte-targeting peptide (AS1) conjugated to lipid nanoparticles and encapsulating siRNA targeting the gene of interest. This strategy has been demonstrated in a mouse model of stroke, in which an experimental treatment was administered intravenously to the model animals. However, the described intervention targeted MEGF10, a key molecule mediating astrocytic phagocytosis of synapses, which is remarkably upregulated during the chronic phase of stroke, leading to synapse loss and exacerbating brain injury. Despite the confirmed high biocompatibility of the used siMEGF10-LNP@AS1 system, further studies are necessary to confirm its safety and the long-term effect of this newly proposed astrocyte-targeted delivery approach [247].

As briefly discussed, cell reprogramming offers a novel approach to enhance their antioxidative potential for therapeutic interventions following CNS injury. Besides the abovementioned delivery strategies, the introduction of antioxidant-encoding transgenes to astrocytes or neurons, such as Nrf2, can be achieved using viral vectors, including lentivirus or adeno-associated virus (AAV) [248,249,250,251]. However, a challenging issue is that prolonged Nrf2 activation in astrocytes following CNS injury poses a potentially significant biological liability despite its well-established neuroprotective effects. Sustained Nrf2 signalling can drive persistent induction of antioxidant and detoxifying enzymes, but it may also disrupt astrocyte homeostasis through maladaptive metabolic reprogramming and impaired autophagic flux. In particular, chronic Nrf2 activation, associated with p62–Keap1 feedback loops, has been linked across multiple tissues to autophagy dysfunction, aberrant cell survival, and reduced capacity to resolve oxidative damage once the acute phase has passed [252]. Moreover, constitutive Nrf2 activity is recognized in cancer biology as a facilitator of prosurvival, anti-apoptotic phenotypes, raising theoretical concerns that long-term upregulation in reactive astrocytes, cells capable of proliferation under injury conditions, could potentiate pathological gliosis or, in extreme scenarios, contribute to tumour-promoting environments [253,254]. Given these risks, astrocyte-targeted Nrf2-based therapies must balance the short-term benefits of oxidative stress reduction with mechanisms ensuring transient, tightly regulated activation to avoid undermining long-term tissue stability and recovery.

Preclinical studies show improved cellular survival, reduced oxidative stress markers, and functional recovery with astrocyte-targeted Nrf2 modulation or modulation of antioxidant genes. To date, these strategies remain preclinical, and no registered human trials specifically delivering antioxidant genes to astrocytes have reported results. It needs further elucidation focused on safety, durable expression, and cell-specific targeting before clinical translation. Key challenges include effective CNS delivery of nucleic acid therapeutics beyond the BBB [255]. While mechanistically promising, clinical translation remains limited, requiring further development of delivery methods and safety profiles.

## 8. Conclusions

Astrocytes are crucial for maintaining redox balance and supporting neuronal survival after CNS injury. Strategies aimed at restoring astrocytic redox homeostasis, such as small-molecule Nrf2 activators, thiol replenishment, and MTAs, hold significant potential to reduce oxidative and inflammatory damage. Additionally, emerging techniques such as astrocyte reprogramming and mRNA, miRNA, siRNA, and gene therapeutics hold promise for neural repair by modulating astrocyte function. Despite promising preclinical results, clinical translation faces challenges related to CNS-specific delivery and long-term safety concerns. Future advances will require improved targeted delivery methods, controlled gene expression, and validation in clinical trials to fully realize the therapeutic potential of astrocytes in re-establishing redox balance after CNS injury. Despite extensive preclinical validation, several barriers constrain the translational feasibility of astrocyte-targeted antioxidative therapies. The BBB penetration remains a major pharmacological challenge, limiting the bioavailability of many small-molecule and gene-based agents. Furthermore, sustained Nrf2 activation, although protective, may cause adverse metabolic reprogramming or interfere with normal redox signalling during long-term administration. In addition, pharmacokinetic heterogeneity across delivery routes and disease contexts complicates dose optimization. Addressing these issues will require innovative delivery systems capable of cell-specific targeting, transient activation kinetics, and integration with biomarkers that monitor CNS redox states in vivo.

## Data Availability

No new data were created or analyzed in this study. Data sharing is not applicable to this article.

## Abbreviations

The following abbreviations are used in this manuscript:

AAV	Adeno-Associated Virus
AD	Alzheimer’s Disease
ALS	Amyotrophic Lateral Sclerosis
AP-1	Activator Protein 1
ApoE	Apolipoprotein E
ARE	Antioxidant Response Element
ATF4	Activating Transcription Factor 4
ATP	Adenosine Triphosphate
Aβ	Amyloid Beta Peptide
BBB	Blood–Brain Barrier
BDNF	Brain-Derived Neurotrophic Factor
bFGF	Basic Fibroblast Growth Factor
C1q	Complement Component 1q
Ca^2+^	Calcium Ion
CAT	Catalase
CBP	CREB-Binding Protein
CHD6	Chromo-ATPase/Helicase DNA-Binding Protein 6
CNS	Central Nervous System
CNTF	Ciliary Neurotrophic Factor
CREB	cAMP Response Element-Binding Protein
CUL3	Cullin 3
DAMPs	Damage-Associated Molecular Patterns
DT-13	Saponin Compound from Liriope muscari
EAAT	Excitatory Amino Acid Transporter
ERK	Extracellular Signal-Regulated Kinase
GCL	γ-Glutamate-Cysteine Ligase
GCLC	γ-Glutamate-Cysteine Ligase Catalytic Subunit
GCLM	γ-Glutamate-Cysteine Ligase Modifier Subunit
GDNF	Glial Cell Line–Derived Neurotrophic Factor
GLT-1	Glutamate Transporter-1
GPx	Glutathione Peroxidase
GSH	Glutathione (reduced form)
GSK-3	Glycogen Synthase Kinase 3
GSSG	Glutathione Disulfide (oxidized form)
GST	Glutathione S-Transferase
HD	Huntington’s Disease
HO-1	Heme Oxygenase-1
IL-1β,3,4,10,33	Interleukin-1β,3,4,10,33
JAK/STAT	Janus Kinase/Signal Transducer and Activator of Transcription
JNK	c-Jun N-terminal Kinase
Keap1	Kelch-Like ECH-Associated Protein 1
MAF	Musculoaponeurotic Fibrosarcoma
MAPK	Mitogen-Activated Protein Kinase
mAβ	Monomeric Amyloid Beta
miRNA	MicroRNA
Mito-Q	Mitoquinone (ubiquinone derivative)
Mito-CP	Mito-Carboxy Proxyl
Mrp1, 5	Multidrug Resistance Protein 1, 5
MTA	Mitochondria-Targeted Antioxidant
NAC	N-Acetylcysteine
NADPH	Nicotinamide Adenine Dinucleotide Phosphate (reduced form)
NF-κB	Nuclear Factor Kappa B
NGF	Nerve Growth Factor
NOX2	NADPH Oxidase 2
NQO1	NAD(P)H Quinone Oxidoreductase 1
Nrf2	Nuclear Factor Erythroid 2-Related Factor 2
PD	Parkinson’s Disease
PDGF	Platelet-Derived Growth Factor
PKA	Protein Kinase A
PKC	Protein Kinase C
PPP	Pentose Phosphate Pathway
Prx	Peroxiredoxin
RAGE	Receptor for Advanced Glycation End-Products
Rg1/Rh1/Rb1	Ginsenosides Rg1, Rh1, Rb1 (ginseng-derived saponins)
RIPK3	Receptor-Interacting Serine/Threonine-Protein Kinase 3
RNS	Reactive Nitrogen Species
ROS	Reactive Oxygen Species
RXRα	Retinoic X Receptor Alpha
SCI	Spinal Cord Injury
siRNA	Small Interfering RNA
SkQ1	Plastoquinone-Derivative Antioxidant
sMAF	Small MAF Protein (MAFG/MAFK)
SOD	Superoxide Dismutase
SQSTM1/p62	Sequestosome 1 Gene Encoding Protein p62
SS-31	Elamipretide (Szeto–Schiller peptide 31)
tBHQ	Tert-Butylhydroquinone
TBI	Traumatic Brain Injury
TGFβ	Transforming Growth Factor Beta
TNF-α	Tumor Necrosis Factor Alpha
Trx	Thioredoxin
TrxR	Thioredoxin Reductase
Wnt	Wingless/Integrated signalling patway
xc^−^	Cystine/Glutamate Antiporter
α-Syn	Alpha-Synuclein
β-TrCP	β-Transducing Repeat Containing Protein

## References

[1] Pathak D., Sriram K. (2023). Neuron-Astrocyte Omnidirectional Signaling in Neurological Health and Disease. Front. Mol. Neurosci..

[2] Huang M., Long A., Hao L., Shi Z., Zhang M. (2025). Astrocyte in Neurological Disease: Pathogenesis and Therapy. MedComm.

[3] Won W., Bhalla M., Lee J.H., Lee C.J. (2025). Astrocytes as Key Regulators of Neural Signaling in Health and Disease. Annu. Rev. Neurosci..

[4] Liu T., Rong Z., Li J., Wu H., Wei J. (2025). Three-Dimensional Interactive Network: Mitochondrial-Metabolic-Calcium Homeostasis Driving Alzheimer’s Disease. Genes Dis..

[5] Guo C.Y., Sun L., Chen X.P., Zhang D.S. (2013). Oxidative Stress, Mitochondrial Damage and Neurodegenerative Diseases. Neural Regen. Res..

[6] Beard E., Lengacher S., Dias S., Magistretti P.J., Finsterwald C. (2022). Astrocytes as Key Regulators of Brain Energy Metabolism: New Therapeutic Perspectives. Front. Physiol..

[7] Zhang Y.M., Qi Y.B., Gao Y.N., Chen W.G., Zhou T., Zang Y., Li J. (2023). Astrocyte Metabolism and Signaling Pathways in the CNS. Front. Neurosci..

[8] Vargas M.R., Johnson J.A. (2009). The Nrf2-ARE Cytoprotective Pathway in Astrocytes. Expert Rev. Mol. Med..

[9] Todd A.C., Hardingham G.E. (2020). The Regulation of Astrocytic Glutamate Transporters in Health and Neurodegenerative Diseases. Int. J. Mol. Sci..

[10] Achzet L.M., Davison C.J., Shea M., Sturgeon I., Jackson D.A. (2021). Oxidative Stress Underlies the Ischemia/Reperfusion-Induced Internalization and Degradation of AMPA Receptors. Int. J. Mol. Sci..

[11] Jurcau A., Ardelean I.A. (2021). Molecular Pathophysiological Mechanisms of Ischemia/Reperfusion Injuries after Recanalization Therapy for Acute Ischemic Stroke. J. Integr. Neurosci..

[12] Belov Kirdajova D., Kriska J., Tureckova J., Anderova M. (2020). Ischemia-Triggered Glutamate Excitotoxicity from the Perspective of Glial Cells. Front. Cell. Neurosci..

[13] Hasan A.R., Tasnim F., Aktaruzzaman M., Islam M.T., Rayhan R., Brishti A., Hur J., Porter J.E., Raihan M.O. (2024). The Alteration of Microglial Calcium Homeostasis in Central Nervous System Disorders: A Comprehensive Review. Neuroglia.

[14] Zhang H., Zhang X., Chai Y., Wang Y., Zhang J., Chen X. (2025). Astrocyte-Mediated Inflammatory Responses in Traumatic Brain Injury: Mechanisms and Potential Interventions. Front. Immunol..

[15] Wilson D.M., Cookson M.R., Van Den Bosch L., Zetterberg H., Holtzman D.M., Dewachter I. (2023). Hallmarks of Neurodegenerative Diseases. Cell.

[16] Percário S., Da Silva Barbosa A., Varela E.L.P., Gomes A.R.Q., Ferreira M.E.S., De Nazaré Araújo Moreira T., Dolabela M.F. (2020). Oxidative Stress in Parkinson’s Disease: Potential Benefits of Antioxidant Supplementation. Oxidative Med. Cell. Longev..

[17] Abramov A.Y., Canevari L., Duchen M.R. (2004). β-Amyloid Peptides Induce Mitochondrial Dysfunction and Oxidative Stress in Astrocytes and Death of Neurons through Activation of NADPH Oxidase. J. Neurosci..

[18] Sochocka M., Diniz B.S., Leszek J. (2017). Inflammatory Response in the CNS: Friend or Foe?. Mol. Neurobiol..

[19] Fischer R., Maier O. (2015). Interrelation of Oxidative Stress and Inflammation in Neurodegenerative Disease: Role of TNF. Oxidative Med. Cell. Longev..

[20] Baxter P.S., Hardingham G.E. (2016). Adaptive Regulation of the Brain’s Antioxidant Defences by Neurons and Astrocytes. Free Radic. Biol. Med..

[21] Buendia I., Michalska P., Navarro E., Gameiro I., Egea J., León R. (2016). Nrf2-ARE Pathway: An Emerging Target against Oxidative Stress and Neuroinflammation in Neurodegenerative Diseases. Pharmacol. Ther..

[22] Gote S., Dubey S., Nargund S.L., Thapa S. (2025). A Systematic Review of Natural Products Targeting Nrf2-Keap1-ARE Pathway and Their Influence on Neurodegenerative Disorders. Inflammopharmacology.

[23] Sengoku T., Shiina M., Suzuki K., Hamada K., Sato K., Uchiyama A., Kobayashi S., Oguni A., Itaya H., Kasahara K. (2022). Structural Basis of Transcription Regulation by CNC Family Transcription Factor, Nrf2. Nucleic Acids Res..

[24] Jaramillo M.C., Zhang D.D. (2013). The Emerging Role of the Nrf2-Keap1 Signaling Pathway in Cancer. Genes Dev..

[25] Suzuki T., Motohashi H., Yamamoto M. (2013). Toward Clinical Application of the Keap1–Nrf2 Pathway. Trends Pharmacol. Sci..

[26] Hayes J.D., McMahon M., Chowdhry S., Dinkova-Kostova A.T. (2010). Cancer Chemoprevention Mechanisms Mediated Through the Keap1–Nrf2 Pathway. Antioxid. Redox Signal..

[27] Zipper L.M., Timothy Mulcahy R. (2002). The Keap1 BTB/POZ Dimerization Function Is Required to Sequester Nrf2 in Cytoplasm. J. Biol. Chem..

[28] Lee S., Hu L. (2020). Nrf2 Activation through the Inhibition of Keap1-Nrf2 Protein-Protein Interaction. Med. Chem. Res..

[29] Li X., Zhang D., Hannink M., Beamer L.J. (2004). Crystal Structure of the Kelch Domain of Human Keap1. J. Biol. Chem..

[30] Dinkova-Kostova A.T., Kostov R.V., Canning P. (2017). Keap1, the Cysteine-Based Mammalian Intracellular Sensor for Electrophiles and Oxidants. Arch. Biochem. Biophys..

[31] Dayalan Naidu S., Dinkova-Kostova A.T. (2020). KEAP1, a Cysteine-Based Sensor and a Drug Target for the Prevention and Treatment of Chronic Disease. Open Biol..

[32] Hayes J.D., Dinkova-Kostova A.T. (2014). The Nrf2 Regulatory Network Provides an Interface between Redox and Intermediary Metabolism. Trends Biochem. Sci..

[33] Barrera-Rodríguez R. (2018). Importance of the Keap1-Nrf2 Pathway in NSCLC: Is it a Possible Biomarker?. Biomed. Rep..

[34] Li W., Yu S., Liu T., Kim J.H., Blank V., Li H., Kong A.N.T. (2008). Heterodimerization with Small Maf Proteins Enhances Nuclear Retention of Nrf2 via Masking the NESzip Motif. Biochim. Biophys. Acta Mol. Cell Res..

[35] Baird P.N., Saw S.M., Lanca C., Guggenheim J.A., Smith E.L., Zhou X., Matsui K.O., Wu P.C., Sankaridurg P., Chia A. (2020). Myopia. Nat. Rev. Dis. Primers.

[36] Kobayashi M., Yamamoto M. (2006). Nrf2–Keap1 Regulation of Cellular Defense Mechanisms against Electrophiles and Reactive Oxygen Species. Adv. Enzym. Regul..

[37] Liu T., Lv Y.F., Zhao J.L., You Q.D., Jiang Z.Y. (2021). Regulation of Nrf2 by Phosphorylation: Consequences for Biological Function and Therapeutic Implications. Free Radic. Biol. Med..

[38] Cuadrado A. (2015). Structural and Functional Characterization of Nrf2 Degradation by Glycogen Synthase Kinase 3/β-TrCP. Free Radic. Biol. Med..

[39] Lee J.M., Calkins M.J., Chan K., Kan Y.W., Johnson J.A. (2003). Identification of the NF-E2-Related Factor-2-Dependent Genes Conferring Protection against Oxidative Stress in Primary Cortical Astrocytes Using Oligonucleotide Microarray Analysis. J. Biol. Chem..

[40] Pajares M., Jiménez-Moreno N., García-Yagüe Á.J., Escoll M., de Ceballos M.L., Van Leuven F., Rábano A., Yamamoto M., Rojo A.I., Cuadrado A. (2016). Transcription Factor NFE2L2/NRF2 Is a Regulator of Macroautophagy Genes. Autophagy.

[41] Liberto C.M., Albrecht P.J., Herx L.M., Yong V.W., Levison S.W. (2004). Pro-Regenerative Properties of Cytokine-Activated Astrocytes. J. Neurochem..

[42] Cabezas R., Avila-Rodriguez M., Vega-Vela N.E., Echeverria V., González J., Hidalgo O.A., Santos A.B., Aliev G., Barreto G.E. (2016). Growth Factors and Astrocytes Metabolism: Possible Roles for Platelet Derived Growth Factor. Med. Chem..

[43] Liu B., Teschemacher A.G., Kasparov S. (2017). Neuroprotective Potential of Astroglia. J. Neurosci. Res..

[44] Bélanger M., Magistretti P.J. (2009). The Role of Astroglia in Neuroprotection. Dialogues Clin. Neurosci..

[45] Dringen R., Kussmaul L., Gutterer J.M., Hirrlinger J., Hamprecht B. (1999). The Glutathione System of Peroxide Detoxification is Less Efficient in Neurons than in Astroglial Cells. J. Neurochem..

[46] McBean G.J. (2017). Cysteine, Glutathione, and Thiol Redox Balance in Astrocytes. Antioxidants.

[47] Chen Y., Vartiainen N.E., Ying W., Chan P.H., Koistinaho J., Swanson R.A. (2001). Astrocytes Protect Neurons from Nitric Oxide Toxicity by a Glutathione-Dependent Mechanism. J. Neurochem..

[48] Chen Y., Qin C., Huang J., Tang X., Liu C., Huang K., Xu J., Guo G., Tong A., Zhou L. (2020). The Role of Astrocytes in Oxidative Stress of Central Nervous System: A Mixed Blessing. Cell Prolif..

[49] Lee K.H., Cha M., Lee B.H. (2020). Neuroprotective Effect of Antioxidants in the Brain. Int. J. Mol. Sci..

[50] Castro M.A., Beltrán F.A., Brauchi S., Concha I.I. (2009). A Metabolic Switch in Brain: Glucose and Lactate Metabolism Modulation by Ascorbic Acid. J. Neurochem..

[51] Castro M.A., Pozo M., Cortés C., García M.D.L.A., Concha I.I., Nualart F. (2007). Intracellular Ascorbic Acid Inhibits Transport of Glucose by Neurons, but Not by Astrocytes. J. Neurochem..

[52] Salazar K., Espinoza F., Cerda-Gallardo G., Ferrada L., Magdalena R., Ramírez E., Ulloa V., Saldivia N., Troncoso N., Oviedo M.J. (2021). SVCT2 Overexpression and Ascorbic Acid Uptake Increase Cortical Neuron Differentiation, Which is Dependent on Vitamin C Recycling between Neurons and Astrocytes. Antioxidants.

[53] Miyazaki I., Asanuma M. (2023). Multifunctional Metallothioneins as a Target for Neuroprotection in Parkinson’s Disease. Antioxidants.

[54] Li Z.-D., Kang S., Li H., Yu P., Xie R., Li C., Jing Q., Gong Z., Li L., Li Z. (2025). Absence of Astrocytic Ceruloplasmin Reverses the Senescence Process with Aging of Learning and Memory Abilities. Redox Biol..

[55] Jimenez-Blasco D., Santofimia-Castanõ P., Gonzalez A., Almeida A., Bolanõs J.P. (2015). Astrocyte NMDA Receptors’ Activity Sustains Neuronal Survival through a Cdk5-Nrf2 Pathway. Cell Death Differ..

[56] Bell K.F.S., Al-Mubarak B., Martel M.A., McKay S., Wheelan N., Hasel P., Márkus N.M., Baxter P., Deighton R.F., Serio A. (2015). Neuronal Development is Promoted by Weakened Intrinsic Antioxidant Defences Due to Epigenetic Repression of Nrf2. Nat. Commun..

[57] Baxter P.S., Márkus N.M., Dando O., He X., Al-Mubarak B.R., Qiu J., Hardingham G.E. (2021). Targeted De-Repression of Neuronal Nrf2 Inhibits α-Synuclein Accumulation. Cell Death Dis..

[58] Wilson C., Muñoz-Palma E., González-Billault C. (2018). From Birth to Death: A Role for Reactive Oxygen Species in Neuronal Development. Semin. Cell Dev. Biol..

[59] Bórquez D.A., Urrutia P.J., Wilson C., Van Zundert B., Núñez M.T., González-Billault C. (2016). Dissecting the Role of Redox Signaling in Neuronal Development. J. Neurochem..

[60] Funato Y., Michiue T., Asashima M., Miki H. (2006). The Thioredoxin-Related Redox-Regulating Protein Nucleoredoxin Inhibits Wnt–β-Catenin Signalling through Dishevelled. Nat. Cell Biol..

[61] Rharass T., Lemcke H., Lantow M., Kuznetsov S.A., Weiss D.G., Panáková D. (2014). Ca^2+^-Mediated Mitochondrial Reactive Oxygen Species Metabolism Augments Wnt/β-Catenin Pathway Activation to Facilitate Cell Differentiation. J. Biol. Chem..

[62] Yu X., Malenka R.C. (2003). β-Catenin Is Critical for Dendritic Morphogenesis. Nat. Neurosci..

[63] Rosso S.B., Sussman D., Wynshaw-Boris A., Salinas P.C. (2005). Wnt Signaling through Dishevelled, Rac and JNK Regulates Dendritic Development. Nat. Neurosci..

[64] Yang Y., Higashimori H., Morel L. (2013). Developmental Maturation of Astrocytes and Pathogenesis of Neurodevelopmental Disorders. J. Neurodev. Disord..

[65] Li J., Pan L., Pembroke W.G., Rexach J.E., Godoy M.I., Condro M.C., Alvarado A.G., Harteni M., Chen Y.W., Stiles L. (2021). Conservation and Divergence of Vulnerability and Responses to Stressors between Human and Mouse Astrocytes. Nat. Commun..

[66] Aoyama K. (2021). Glutathione in the Brain. Int. J. Mol. Sci..

[67] Almeida A., Jimenez-Blasco D., Bolaños J.P. (2023). Cross-Talk between Energy and Redox Metabolism in Astrocyte-Neuron Functional Cooperation. Essays Biochem..

[68] Mattson M.P., Liu D. (2002). Energetics and Oxidative Stress in Synaptic Plasticity and Neurodegenerative Disorders. Neuromolecular Med..

[69] Cai Q., Sheng Z.H. (2009). Molecular Motors and Synaptic Assembly. Neuroscientist.

[70] Almeida A., Bolaños J.P. (2001). A Transient Inhibition of Mitochondrial ATP Synthesis by Nitric Oxide Synthase Activation Triggered Apoptosis in Primary Cortical Neurons. J. Neurochem..

[71] Bolaños J.P. (2016). Bioenergetics and Redox Adaptations of Astrocytes to Neuronal Activity. J. Neurochem..

[72] Herrero-Mendez A., Almeida A., Fernández E., Maestre C., Moncada S., Bolaños J.P. (2009). The Bioenergetic and Antioxidant Status of Neurons Is Controlled by Continuous Degradation of a Key Glycolytic Enzyme by APC/C-Cdh1. Nat. Cell Biol..

[73] Bouzier-Sore A.K., Bolaños J.P. (2015). Uncertainties in Pentose-Phosphate Pathway Flux Assessment Underestimate its Contribution to Neuronal Glucose Consumption: Relevance for Neurodegeneration and Aging. Front. Aging Neurosci..

[74] Dringen R., Pfeiffer B., Hamprecht B. (1999). Synthesis of the Antioxidant Glutathione in Neurons: Supply by Astrocytes of CysGly as Precursor for Neuronal Glutathione. J. Neurosci..

[75] Quintana-Cabrera R., Bolaños J.P. (2013). Glutathione and γ-Glutamylcysteine in Hydrogen Peroxide Detoxification. Methods Enzymol..

[76] Fernandez-Fernandez S., Almeida A., Bolaños J.P. (2012). Antioxidant and Bioenergetic Coupling between Neurons and Astrocytes. Biochem. J..

[77] Minich T., Riemer J., Schulz J.B., Wielinga P., Wijnholds J., Dringen R. (2006). The Multidrug Resistance Protein 1 (Mrp1), but Not Mrp5, Mediates Export of Glutathione and Glutathione Disulfide from Brain Astrocytes. J. Neurochem..

[78] Bell K.F.S., Fowler J.H., Al-Mubarak B., Horsburgh K., Hardingham G.E. (2011). Activation of Nrf2-Regulated Glutathione Pathway Genes by Ischemic Preconditioning. Oxidative Med. Cell. Longev..

[79] Shih A.Y., Johnson D.A., Wong G., Kraft A.D., Jiang L., Erb H., Johnson J.A., Murphy T.H. (2003). Coordinate Regulation of Glutathione Biosynthesis and Release by Nrf2-Expressing Glia Potently Protects Neurons from Oxidative Stress. J. Neurosci..

[80] Davis C.H.O., Kim K.Y., Bushong E.A., Mills E.A., Boassa D., Shih T., Kinebuchi M., Phan S., Zhou Y., Bihlmeyer N.A. (2014). Transcellular Degradation of Axonal Mitochondria. Proc. Natl. Acad. Sci. USA.

[81] Hayakawa K., Esposito E., Wang X., Terasaki Y., Liu Y., Xing C., Ji X., Lo E.H. (2016). Transfer of Mitochondria from Astrocytes to Neurons after Stroke. Nature.

[82] Berridge M.V., Schneider R.T., McConnell M.J. (2016). Mitochondrial Transfer from Astrocytes to Neurons Following Ischemic Insult: Guilt by Association?. Cell Metab..

[83] Ioannou M.S., Jackson J., Sheu S.H., Chang C.L., Weigel A.V., Liu H., Pasolli H.A., Xu C.S., Pang S., Matthies D. (2019). Neuron-Astrocyte Metabolic Coupling Protects against Activity-Induced Fatty Acid Toxicity. Cell.

[84] Qiu J., Dando O., Febery J.A., Fowler J.H., Chandran S., Hardingham G.E. (2020). Neuronal Activity and Its Role in Controlling Antioxidant Genes. Int. J. Mol. Sci..

[85] Hasel P., Dando O., Jiwaji Z., Baxter P., Todd A.C., Heron S., Márkus N.M., McQueen J., Hampton D.W., Torvell M. (2017). Neurons and Neuronal Activity Control Gene Expression in Astrocytes to Regulate Their Development and Metabolism. Nat. Commun..

[86] Habas A., Hahn J., Wang X., Margeta M. (2013). Neuronal Activity Regulates Astrocytic Nrf2 Signaling. Proc. Natl. Acad. Sci. USA.

[87] Papadia S., Soriano F.X., Léveillé F., Martel M.A., Dakin K.A., Hansen H.H., Kaindl A., Sifringer M., Fowler J., Stefovska V. (2008). Synaptic NMDA Receptor Activity Boosts Intrinsic Antioxidant Defenses. Nat. Neurosci..

[88] Hardingham G.E., Lipton S.A. (2011). Regulation of Neuronal Oxidative and Nitrosative Stress by Endogenous Protective Pathways and Disease Processes. Antioxid. Redox Signal..

[89] Nguyen T., Yang C.S., Pickett C.B. (2004). The Pathways and Molecular Mechanisms Regulating Nrf2 Activation in Response to Chemical Stress. Free Radic. Biol. Med..

[90] Mayorquin L.C., Rodriguez A.V., Sutachan J.J., Albarracín S.L. (2018). Connexin-Mediated Functional and Metabolic Coupling Between Astrocytes and Neurons. Front. Mol. Neurosci..

[91] Jiwaji Z., Hardingham G.E. (2023). The Consequences of Neurodegenerative Disease on Neuron-Astrocyte Metabolic and Redox Interactions. Neurobiol. Dis..

[92] Schipke C.G., Kettenmann H. (2004). Astrocyte Responses to Neuronal Activity. Glia.

[93] Fellin T., Carmignoto G. (2004). Neurone-to-Astrocyte Signalling in the Brain Represents a Distinct Multifunctional Unit. J. Physiol..

[94] Parpura V., Basarsky T.A., Liu F., Jeftinija K., Jeftinija S., Haydon P.G. (1994). Glutamate-Mediated Astrocyte–Neuron Signalling. Nature.

[95] Sofroniew M.V. (2009). Molecular Dissection of Reactive Astrogliosis and Glial Scar Formation. Trends Neurosci..

[96] Rosales-Corral S., Reiter R.J., Tan D.X., Ortiz G.G., Lopez-Armas G. (2010). Functional Aspects of Redox Control during Neuroinflammation. Antioxid. Redox Signal..

[97] Butterfield D.A. (2015). Redox Signaling in Neurodegeneration. Neurobiol. Dis..

[98] Aguilera G., Colín-González A.L., Rangel-López E., Chavarría A., Santamaría A. (2018). Redox Signaling, Neuroinflammation, and Neurodegeneration. Antioxid. Redox Signal..

[99] Franco R., Vargas M.R. (2018). Redox Biology in Neurological Function, Dysfunction, and Aging. Antioxid. Redox Signal..

[100] Spiers J.G., Chen H.J.C., Steinert J.R. (2023). Redox Mechanisms and Their Pathological Role in Prion Diseases: The Road to Ruin. PLoS Pathog..

[101] Raivich G., Bohatschek M., Kloss C.U.A., Werner A., Jones L.L., Kreutzberg G.W. (1999). Neuroglial Activation Repertoire in the Injured Brain: Graded Response, Molecular Mechanisms and Cues to Physiological Function. Brain Res. Rev..

[102] Guo X., Kang J., Wang Z., Wang Y., Liu M., Zhu D., Yang F., Kang X. (2022). Nrf2 Signaling in the Oxidative Stress Response After Spinal Cord Injury. Neuroscience.

[103] Dugue R., Nath M., Dugue A., Barone F.C. (2017). Roles of Pro- and Anti-Inflammatory Cytokines in Traumatic Brain Injury and Acute Ischemic Stroke. Mechanisms of Neuroinflammation.

[104] Jin W., Wang H., Yan W., Xu L., Wang X., Zhao X., Yang X., Chen G., Ji Y. (2008). Disruption of Nrf2 Enhances Upregulation of Nuclear Factor-ΚB Activity, Proinflammatory Cytokines, and Intercellular Adhesion Molecule-1 in the Brain after Traumatic Brain Injury. Mediat. Inflamm..

[105] Samarghandian S., Pourbagher-Shahri A.M., Ashrafizadeh M., Khan H., Forouzanfar F., Aramjoo H., Farkhondeh T. (2020). A Pivotal Role of the Nrf2 Signaling Pathway in Spinal Cord Injury: A Prospective Therapeutics Study. CNS Neurol. Disord. Drug Targets.

[106] Silvestro S., Mazzon E. (2022). Nrf2 Activation: Involvement in Central Nervous System Traumatic Injuries. A Promising Therapeutic Target of Natural Compounds. Int. J. Mol. Sci..

[107] Heurtaux T., Bouvier D.S., Benani A., Helgueta Romero S., Frauenknecht K.B.M., Mittelbronn M., Sinkkonen L. (2022). Normal and Pathological NRF2 Signalling in the Central Nervous System. Antioxidants.

[108] Fadoul G., Ikonomovic M., Zhang F., Yang T. (2024). The Cell-Specific Roles of Nrf2 in Acute and Chronic Phases of Ischemic Stroke. CNS Neurosci. Ther..

[109] Sivandzade F., Prasad S., Bhalerao A., Cucullo L. (2019). NRF2 and NF-ҚB Interplay in Cerebrovascular and Neurodegenerative Disorders: Molecular Mechanisms and Possible Therapeutic Approaches. Redox Biol..

[110] Chandran R., Kim T.H., Mehta S.L., Udho E., Chanana V., Cengiz P., Kim H.W., Kim C., Vemuganti R. (2018). A Combination Antioxidant Therapy to Inhibit NOX2 and Activate Nrf2 Decreases Secondary Brain Damage and Improves Functional Recovery after Traumatic Brain Injury. J. Cereb. Blood Flow Metab..

[111] Muneer P.M.A. (2023). Nrf2 as a Potential Therapeutic Target for Traumatic Brain Injury. J. Integr. Neurosci..

[112] Liu W., Tang Y., Feng J. (2011). Cross Talk between Activation of Microglia and Astrocytes in Pathological Conditions in the Central Nervous System. Life Sci..

[113] Liddelow S.A., Guttenplan K.A., Clarke L.E., Bennett F.C., Bohlen C.J., Schirmer L., Bennett M.L., Münch A.E., Chung W.S., Peterson T.C. (2017). Neurotoxic Reactive Astrocytes Are Induced by Activated Microglia. Nature.

[114] Li J., Wang X., Qin S. (2021). Molecular Mechanisms and Signaling Pathways of Reactive Astrocytes Responding to Traumatic Brain Injury. Histol. Histopathol..

[115] He Y., Liu X., Chen Z. (2020). Glial Scar—A Promising Target for Improving Outcomes After CNS Injury. J. Mol. Neurosci..

[116] Gao Z., Zhu Q., Zhang Y., Zhao Y., Cai L., Shields C.B., Cai J. (2013). Reciprocal Modulation between Microglia and Astrocyte in Reactive Gliosis Following the CNS Injury. Mol. Neurobiol..

[117] Kigerl K.A., Ankeny D.P., Garg S.K., Wei P., Guan Z., Lai W., Mctigue D.M., Banerjee R., Popovich P.G. (2011). System X_c_^−^ Regulates Microglia and Macrophage Glutamate Excitotoxicity In Vivo. Exp. Neurol..

[118] Guerriero R.M., Giza C.C., Rotenberg A. (2015). Glutamate and GABA Imbalance Following Traumatic Brain Injury. Curr. Neurol. Neurosci. Rep..

[119] Aizenman E., Loring R.H., Reynolds I.J., Rosenberg P.A. (2020). The Redox Biology of Excitotoxic Processes: The NMDA Receptor, TOPA Quinone, and the Oxidative Liberation of Intracellular Zinc. Front. Neurosci..

[120] Ma M., Jiang W., Zhou R. (2024). DAMPs and DAMP-Sensing Receptors in Inflammation and Diseases. Immunity.

[121] Chang N.P., DaPrano E.M., Lindman M., Estevez I., Chou T.W., Evans W.R., Nissenbaum M., McCourt M., Alzate D., Atkins C. (2024). Neuronal DAMPs Exacerbate Neurodegeneration via Astrocytic RIPK3 Signaling. JCI Insight.

[122] Reyes R.C., Brennan A.M., Shen Y., Baldwin Y., Swanson R.A. (2012). Activation of Neuronal NMDA Receptors Induces Superoxide-Mediated Oxidative Stress in Neighboring Neurons and Astrocytes. J. Neurosci..

[123] Haskew-Layton R.E., Payappilly J.B., Smirnova N.A., Ma T.C., Chan K.K., Murphy T.H., Guo H., Langley B., Sultana R., Butterfield D.A. (2010). Controlled Enzymatic Production of Astrocytic Hydrogen Peroxide Protects Neurons from Oxidative Stress via an Nrf2-Independent Pathway. Proc. Natl. Acad. Sci. USA.

[124] Huang Q., Zhang H., Chen S., Wang Y., Zhou J. (2025). Ferroptosis in Central Nervous System Injuries: Molecular Mechanisms, Diagnostic Approaches, and Therapeutic Strategies. Front. Cell. Neurosci..

[125] Krajciova G., Filipcik P., Cente M., Skrabana R., Novak M., Shenk J.C., Castellani R.J., Moreira P., Aliev G., Siedlak S.L. (2009). Iron-Induced Oxidative Stress in Primary Culture of Resting and Activated Astrocytes. Alzheimer’s Dement..

[126] Hoepken H.H., Korten T., Robinsont S.R., Dringen R. (2004). Iron Accumulation, Iron-Mediated Toxicity and Altered Levels of Ferritin and Transferrin Receptor in Cultured Astrocytes during Incubation with Ferric Ammonium Citrate. J. Neurochem..

[127] Geppert M., Hohnholt M.C., Nürnberger S., Dringen R. (2012). Ferritin Up-Regulation and Transient ROS Production in Cultured Brain Astrocytes after Loading with Iron Oxide Nanoparticles. Acta Biomater..

[128] Juurlink B.H.J. (1997). Response of Glial Cells to Ischemia: Roles of Reactive Oxygen Species and Glutathione. Neurosci. Biobehav. Rev..

[129] Hohnholt M.C., Dringen R. (2013). Uptake and Metabolism of Iron and Iron Oxide Nanoparticles in Brain Astrocytes. Biochem. Soc. Trans..

[130] Cheli V.T., Correale J., Paez P.M., Pasquini J.M. (2020). Iron Metabolism in Oligodendrocytes and Astrocytes, Implications for Myelination and Remyelination. ASN Neuro.

[131] Li B., Xia M., Zorec R., Parpura V., Verkhratsky A. (2021). Astrocytes in Heavy Metal Neurotoxicity and Neurodegeneration. Brain Res..

[132] David S., Jhelum P., Ryan F., Jeong S.Y., Kroner A. (2021). Dysregulation of Iron Homeostasis in the CNS and the Role of Ferroptosis in Neurodegenerative Disorders. Antioxid. Redox Signal..

[133] Xu S.Y., Ni S.M., Zeng C.L., Peng Y.J. (2023). Role of Ferroptosis in Glial Cells after Ischemic Stroke. Front. Biosci..

[134] Li Y., Xiao D., Wang X. (2022). The Emerging Roles of Ferroptosis in Cells of the Central Nervous System. Front. Neurosci..

[135] Ageeva T., Rizvanov A., Mukhamedshina Y. (2024). NF-ΚB and JAK/STAT Signaling Pathways as Crucial Regulators of Neuroinflammation and Astrocyte Modulation in Spinal Cord Injury. Cells.

[136] Wardyn J.D., Ponsford A.H., Sanderson C.M. (2015). Dissecting Molecular Cross-Talk between Nrf2 and NF-ΚB Response Pathways. Biochem. Soc. Trans..

[137] Bonvento G., Bolaños J.P. (2021). Astrocyte-Neuron Metabolic Cooperation Shapes Brain Activity. Cell Metab..

[138] Bartnik-Olson B.L., Oyoyo U., Hovda D.A., Sutton R.L. (2010). Astrocyte Oxidative Metabolism and Metabolite Trafficking after Fluid Percussion Brain Injury in Adult Rats. J. Neurotrauma.

[139] Burda J.E., Bernstein A.M., Sofroniew M.V. (2016). Astrocyte Roles in Traumatic Brain Injury. Exp. Neurol..

[140] Chen Y., Swanson R.A. (2003). Astrocytes and Brain Injury. J. Cereb. Blood Flow Metab..

[141] von Leden R.E., Parker K.N., Bates A.A., Noble-Haeusslein L.J., Donovan M.H. (2019). The Emerging Role of Neutrophils as Modifiers of Recovery After Traumatic Injury to the Developing Brain. Exp. Neurol..

[142] Morganti-Kossmann M.C., Semple B.D., Hellewell S.C., Bye N., Ziebell J.M. (2019). The Complexity of Neuroinflammation Consequent to Traumatic Brain Injury: From Research Evidence to Potential Treatments. Acta Neuropathol..

[143] Jantzie L., El Demerdash N., Newville J.C., Robinson S. (2019). Time to Reconsider Extended Erythropoietin Treatment for Infantile Traumatic Brain Injury?. Exp. Neurol..

[144] Karve I.P., Taylor J.M., Crack P.J. (2016). The Contribution of Astrocytes and Microglia to Traumatic Brain Injury. Br. J. Pharmacol..

[145] Wang J.F., Li Y., Song J.N., Pang H.G. (2014). Role of Hydrogen Sulfide in Secondary Neuronal Injury. Neurochem. Int..

[146] Ahmed S.M., Rzigalinski B.A., Willoughby K.A., Sitterding H.A., Ellis E.F. (2000). Stretch-Induced Injury Alters Mitochondrial Membrane Potential and Cellular ATP in Cultured Astrocytes and Neurons. J. Neurochem..

[147] Hilkens N.A., Casolla B., Leung T.W., de Leeuw F.E. (2024). Stroke. Lancet.

[148] Xu L., Emery J.F., Ouyang Y.B., Voloboueva L.A., Giffard R.G. (2010). Astrocyte Targeted Overexpression of Hsp72 or SOD2 Reduces Neuronal Vulnerability to Forebrain Ischemia. Glia.

[149] Li L., Stary C.M. (2016). Targeting Glial Mitochondrial Function for Protection from Cerebral Ischemia: Relevance, Mechanisms, and the Role of MicroRNAs. Oxidative Med. Cell. Longev..

[150] Choudhury G.R., Ding S. (2016). Reactive Astrocytes and Therapeutic Potential in Focal Ischemic Stroke. Neurobiol. Dis..

[151] Gouix E., Buisson A., Nieoullon A., Kerkerian-Le Goff L., Tauskela J.S., Blondeau N., Had-Aissouni L. (2014). Oxygen Glucose Deprivation-Induced Astrocyte Dysfunction Provokes Neuronal Death through Oxidative Stress. Pharmacol. Res..

[152] Kurkinen M., Fułek M., Fułek K., Beszłej J.A., Kurpas D., Leszek J. (2023). The Amyloid Cascade Hypothesis in Alzheimer’s Disease: Should We Change Our Thinking?. Biomolecules.

[153] Taniguchi K., Yamamoto F., Amano A., Tamaoka A., Sanjo N., Yokota T., Kametani F., Araki W. (2022). Amyloid-β Oligomers Interact with NMDA Receptors Containing GluN2B Subunits and Metabotropic Glutamate Receptor 1 in Primary Cortical Neurons: Relevance to the Synapse Pathology of Alzheimer’s Disease. Neurosci. Res..

[154] De Felice F.G., Velasco P.T., Lambert M.P., Viola K., Fernandez S.J., Ferreira S.T., Klein W.L. (2007). Aβ Oligomers Induce Neuronal Oxidative Stress through an N-Methyl-D-Aspartate Receptor-Dependent Mechanism that is Blocked by the Alzheimer Drug Memantine. J. Biol. Chem..

[155] Quintana D.D., Garcia J.A., Anantula Y., Rellick S.L., Engler-Chiurazzi E.B., Sarkar S.N., Brown C.M., Simpkins J.W. (2020). Amyloid-β Causes Mitochondrial Dysfunction via a Ca^2+^-Driven Upregulation of Oxidative Phosphorylation and Superoxide Production in Cerebrovascular Endothelial Cells. J. Alzheimer’s Dis..

[156] Cheignon C., Tomas M., Bonnefont-Rousselot D., Faller P., Hureau C., Collin F. (2018). Oxidative Stress and the Amyloid Beta Peptide in Alzheimer’s Disease. Redox Biol..

[157] Ramsey C.P., Glass C.A., Montgomery M.B., Lindl K.A., Ritson G.P., Chia L.A., Hamilton R.L., Chu C.T., Jordan-Sciutto K.L. (2007). Expression of Nrf2 in Neurodegenerative Diseases. J. Neuropathol. Exp. Neurol..

[158] Lastres-Becker I., Innamorato N.G., Jaworski T., Rábano A., Kügler S., Van Leuven F., Cuadrado A. (2014). Fractalkine Activates NRF2/NFE2L2 and Heme Oxygenase 1 to Restrain Tauopathy-Induced Microgliosis. Brain.

[159] Tanji K., Maruyama A., Odagiri S., Mori F., Itoh K., Kakita A., Takahashi H., Wakabayashi K. (2013). Keap1 Is Localized in Neuronal and Glial Cytoplasmic Inclusions in Various Neurodegenerative Diseases. J. Neuropathol. Exp. Neurol..

[160] Raina A.K., Templeton D.J., Deak J.C., Perry G., Smith M.A. (1999). Quinone Reductase (NQO1), a Sensitive Redox Indicator, Is Increased in Alzheimer’s Disease. Redox Rep..

[161] Wang Y., Santa-Cruz K., Decarli C., Johnson J.A. (2000). NAD(P)H:Quinone Oxidoreductase Activity Is Increased in Hippocampal Pyramidal Neurons of Patients with Alzheimer’s Disease. Neurobiol. Aging.

[162] SantaCruz K.S., Yazlovitskaya E., Collins J., Johnson J., DeCarli C. (2004). Regional NAD(P)H:Quinone Oxidoreductase Activity in Alzheimer’s Disease. Neurobiol. Aging.

[163] Schipper H.M., Bennett D.A., Liberman A., Bienias J.L., Schneider J.A., Kelly J., Arvanitakis Z. (2006). Glial Heme Oxygenase-1 Expression in Alzheimer Disease and Mild Cognitive Impairment. Neurobiol. Aging.

[164] Schipper H.M., Cissé S., Stopa E.G. (1995). Expression of Heme Oxygenase-1 in the Senescent and Alzheimer-Diseased Brain. Ann. Neurol..

[165] Smith M.A., Kutty R.K., Richey P.L., Yan S.D., Stern D., Chader G.J., Wiggert B., Petersen R.B., Perry G. (1994). Heme Oxygenase-1 is Associated with the Neurofibrillary Pathology of Alzheimer’s Disease. Am. J. Pathol..

[166] Fiebig C., Keiner S., Ebert B., Schäffner I., Jagasia R., Lie D.C., Beckervordersandforth R. (2019). Mitochondrial Dysfunction in Astrocytes Impairs the Generation of Reactive Astrocytes and Enhances Neuronal Cell Death in the Cortex upon Photothrombotic Lesion. Front. Mol. Neurosci..

[167] Burda J.E., Sofroniew M.V. (2014). Reactive Gliosis and the Multicellular Response to CNS Damage and Disease. Neuron.

[168] Lemoine L., Saint-Aubert L., Nennesmo I., Gillberg P.G., Nordberg A. (2017). Cortical Laminar Tau Deposits and Activated Astrocytes in Alzheimer’s Disease Visualised by 3 H-THK5117 and 3 H-Deprenyl Autoradiography. Sci. Rep..

[169] Marutle A., Gillberg P.G., Bergfors A., Yu W., Ni R., Nennesmo I., Voytenko L., Nordberg A. (2013). 3H-Deprenyl and 3H-PIB Autoradiography Show Different Laminar Distributions of Astroglia and Fibrillar β-Amyloid in Alzheimer Brain. J. Neuroinflamm..

[170] Serrano-Pozo A., Mielke M.L., Gómez-Isla T., Betensky R.A., Growdon J.H., Frosch M.P., Hyman B.T. (2011). Reactive Glia Not Only Associates with Plaques but Also Parallels Tangles in Alzheimer’s Disease. Am. J. Pathol..

[171] Heneka M.T., Rodríguez J.J., Verkhratsky A. (2010). Neuroglia in Neurodegeneration. Brain Res. Rev..

[172] Ye B., Shen H., Zhang J., Zhu Y.G., Ransom B.R., Chen X.C., Ye Z.C. (2015). Dual Pathways Mediate β-Amyloid Stimulated Glutathione Release from Astrocytes. Glia.

[173] Bantle C.M., Hirst W.D., Weihofen A., Shlevkov E. (2021). Mitochondrial Dysfunction in Astrocytes: A Role in Parkinson’s Disease?. Front. Cell Dev. Biol..

[174] Sznejder-Pachołek A., Joniec-Maciejak I., Wawer A., Ciesielska A., Mirowska-Guzel D. (2017). The Effect of α-Synuclein on Gliosis and IL-1α, TNFα, IFNγ, TGFβ Expression in Murine Brain. Pharmacol. Rep..

[175] Valdinocci D., Radford R.A.W., Siow S.M., Chung R.S., Pountney D.L. (2017). Potential Modes of Intercellular α-Synuclein Transmission. Int. J. Mol. Sci..

[176] Song Y.J.C., Halliday G.M., Holton J.L., Lashley T., Osullivan S.S., McCann H., Lees A.J., Ozawa T., Williams D.R., Lockhart P.J. (2009). Degeneration in Different Parkinsonian Syndromes Relates to Astrocyte Type and Astrocyte Protein Expression. J. Neuropathol. Exp. Neurol..

[177] Lee H.J., Suk J.E., Patrick C., Bae E.J., Cho J.H., Rho S., Hwang D., Masliah E., Lee S.J. (2010). Direct Transfer of α-Synuclein from Neuron to Astroglia Causes Inflammatory Responses in Synucleinopathies. J. Biol. Chem..

[178] Fellner L., Irschick R., Schanda K., Reindl M., Klimaschewski L., Poewe W., Wenning G.K., Stefanova N. (2013). Toll-like Receptor 4 is Required for α-Synuclein Dependent Activation of Microglia and Astroglia. Glia.

[179] Chavarría C., Rodríguez-Bottero S., Quijano C., Cassina P., Souza J.M. (2018). Impact of Monomeric, Oligomeric and Fibrillar Alpha-Synuclein on Astrocyte Reactivity and Toxicity to Neurons. Biochem. J..

[180] Angelova P.R., Horrocks M.H., Klenerman D., Gandhi S., Abramov A.Y., Shchepinov M.S. (2015). Lipid Peroxidation is Essential for α-Synuclein-Induced Cell Death. J. Neurochem..

[181] Lee J., Hyeon S.J., Im H., Ryu H., Kim Y., Ryu H. (2016). Astrocytes and Microglia as Non-Cell Autonomous Players in the Pathogenesis of ALS. Exp. Neurobiol..

[182] Ferraiuolo L., Higginbottom A., Heath P.R., Barber S., Greenald D., Kirby J., Shaw P.J. (2011). Dysregulation of Astrocyte-Motoneuron Cross-Talk in Mutant Superoxide Dismutase 1-Related Amyotrophic Lateral Sclerosis. Brain.

[183] Bakshi R., Xu Y., Mueller K.A., Chen X., Granucci E., Paganoni S., Sadri-Vakili G., Schwarzschild M.A. (2018). Urate Mitigates Oxidative Stress and Motor Neuron Toxicity of Astrocytes Derived from ALS-Linked SOD1 G93A Mutant Mice. Mol. Cell. Neurosci..

[184] Kraft A.D., Johnson D.A., Johnson J.A. (2004). Nuclear Factor E2-Related Factor 2-Dependent Antioxidant Response Element Activation by Tert-Butylhydroquinone and Sulforaphane Occurring Preferentially in Astrocytes Conditions Neurons against Oxidative Insult. J. Neurosci..

[185] Zhang Z.W., Liang J., Yan J.X., Ye Y.C., Wang J.J., Chen C., Sun H.T., Chen F., Tu Y., Li X.H. (2020). TBHQ Improved Neurological Recovery after Traumatic Brain Injury by Inhibiting the Overactivation of Astrocytes. Brain Res..

[186] Lu X.Y., Wang H.D., Xu J.G., Ding K., Li T. (2014). Pretreatment with Tert-Butylhydroquinone Attenuates Cerebral Oxidative Stress in Mice after Traumatic Brain Injury. J. Surg. Res..

[187] Jin W., Kong J., Wang H., Wu J., Lu T., Jiang J., Ni H., Liang W. (2011). Protective Effect of Tert-Butylhydroquinone on Cerebral Inflammatory Response Following Traumatic Brain Injury in Mice. Injury.

[188] Khezerlou A., Akhlaghi A.p., Alizadeh A.M., Dehghan P., Maleki P. (2022). Alarming Impact of the Excessive Use of Tert-Butylhydroquinone in Food Products: A Narrative Review. Toxicol. Rep..

[189] Danilov C.A., Chandrasekaran K., Racz J., Soane L., Zielke C., Fiskum G. (2009). Sulforaphane Protects Astrocytes against Oxidative Stress and Delayed Death Caused by Oxygen and Glucose Deprivation. Glia.

[190] Bergström P., Andersson H.C., Gao Y., Karlsson J.O., Nodin C., Anderson M.F., Nilsson M., Hammarsten O. (2011). Repeated Transient Sulforaphane Stimulation in Astrocytes Leads to Prolonged Nrf2-Mediated Gene Expression and Protection from Superoxide-Induced Damage. Neuropharmacology.

[191] Jazwa A., Rojo A.I., Innamorato N.G., Hesse M., Fernández-Ruiz J., Cuadrado A. (2011). Pharmacological Targeting of the Transcription Factor Nrf2 at the Basal Ganglia Provides Disease Modifying Therapy for Experimental Parkinsonism. Antioxid. Redox Signal..

[192] Wang R., Bai H., Yang D., Wuhanqimuge, Bai S., Xiao H., Baigude H., Gao N. (2025). Overexpression of BDNF by Astrocytes Targeted Delivery of MRNA Ameliorates Cognitive Impairment in Mouse Model of TBI. ACS Chem. Neurosci..

[193] Sun J., Hu H., Ren X., Simpkins J.W. (2016). Tert-Butylhydroquinone Compromises Survival in Murine Experimental Stroke. Neurotoxicology Teratol..

[194] Klomparens E.A., Ding Y. (2019). The Neuroprotective Mechanisms and Effects of Sulforaphane. Brain Circ..

[195] Zheng W., Li X., Zhang T., Wang J. (2022). Biological Mechanisms and Clinical Efficacy of Sulforaphane for Mental Disorders. Gen. Psychiatr..

[196] Hu L., Cao Y., Chen H., Xu L., Yang Q., Zhou H., Li J., Yu Q., Dou Z., Li Y. (2022). The Novel Nrf2 Activator Omaveloxolone Regulates Microglia Phenotype and Ameliorates Secondary Brain Injury after Intracerebral Hemorrhage in Mice. Oxidative Med. Cell. Longev..

[197] Abeti R., Baccaro A., Esteras N., Giunti P. (2018). Novel Nrf2-Inducer Prevents Mitochondrial Defects and Oxidative Stress in Friedreich’s Ataxia Models. Front. Cell. Neurosci..

[198] Reisman S.A., Gahir S.S., Lee C.Y.I., Proksch J.W., Sakamoto M., Ward K.W. (2019). Pharmacokinetics and Pharmacodynamics of the Novel Nrf2 Activator Omaveloxolone in Primates. Drug Des. Dev. Ther..

[199] Liu S., Chen W., Zhao Y., Zong Y., Li J., He Z. (2023). Research Progress on Effects of Ginsenoside Rg2 and Rh1 on Nervous System and Related Mechanisms. Molecules.

[200] Chen Y., Evankovich J.W., Lear T.B., Tuncer F., Kennerdell J.R., Camarco D.P., Shishido M.S., Liu Y., Chen B.B. (2020). A Small Molecule NRF2 Activator BC-1901S Ameliorates Inflammation through DCAF1/NRF2 Axis. Redox Biol..

[201] Chu S.-F., Zhang Z., Zhou X., He W.-B., Chen C., Luo P., Liu D.-D., Ai Q.-D., Gong H.-F., Wang Z.-Z. (2019). Ginsenoside Rg1 Protects against Ischemic/Reperfusion-Induced Neuronal Injury through MiR-144/Nrf2/ARE Pathway. Acta Pharmacol. Sin..

[202] Li F., Lv Y.N., Tan Y.S., Shen K., Zhai K.F., Chen H.L., Kou J.P., Yu B.Y. (2015). An Integrated Pathway Interaction Network for the Combination of Four Effective Compounds from ShengMai Preparations in the Treatment of Cardio-Cerebral Ischemic Diseases. Acta Pharmacol. Sin..

[203] Zhao Y.N., Shao X., Ouyang L.F., Chen L., Gu L. (2018). Qualitative Detection of Ginsenosides in Brain Tissues after Oral Administration of High-Purity Ginseng Total Saponins by Using Polyclonal Antibody against Ginsenosides. Chin. J. Nat. Med..

[204] Wang R., Li Y.N., Wang G.J., Hao H.P., Wu X.L., Zhou F. (2009). Neuroprotective Effects and Brain Transport of Ginsenoside Rg1. Chin. J. Nat. Med..

[205] Park J.D. (2024). Metabolism and Drug Interactions of Korean Ginseng Based on the Pharmacokinetic Properties of Ginsenosides: Current Status and Future Perspectives. J. Ginseng Res..

[206] Shieh P., Jan C.R., Liang W.Z. (2019). The Protective Effects of the Antioxidant N-Acetylcysteine (NAC) against Oxidative Stress-Associated Apoptosis Evoked by the Organophosphorus Insecticide Malathion in Normal Human Astrocytes. Toxicology.

[207] Ramaswamy S., Rodriguez A., Driscoll D., Rao V. (2017). Nutraceuticals for Traumatic Brain Injury: Should You Recommend Their Use. Curr. Psychiatry.

[208] Pandya J.D., Readnower R.D., Patel S.P., Yonutas H.M., Pauly J.R., Goldstein G.A., Rabchevsky A.G., Sullivan P.G. (2014). N-Acetylcysteine Amide Confers Neuroprotection, Improves Bioenergetics and Behavioral Outcome Following TBI. Exp. Neurol..

[209] Kawoos U., McCarron R.M., Chavko M. (2017). Protective Effect of N-Acetylcysteine Amide on Blast-Induced Increase in Intracranial Pressure in Rats. Front. Neurol..

[210] Kawoos U., Abutarboush R., Zarriello S., Qadri A., Ahlers S.T., McCarron R.M., Chavko M. (2019). N-Acetylcysteine Amide Ameliorates Blast-Induced Changes in Blood-Brain Barrier Integrity in Rats. Front. Neurol..

[211] Zhou Y., Wang H.D., Zhou X.M., Fang J., Zhu L., Ding K. (2018). N-Acetylcysteine Amide Provides Neuroprotection via Nrf2-ARE Pathway in a Mouse Model of Traumatic Brain Injury. Drug Des. Dev. Ther..

[212] Clark R.S.B., Empey P.E., Kochanek P.M., Bell M.J. (2023). N-Acetylcysteine and Probenecid Adjuvant Therapy for Traumatic Brain Injury. Neurotherapeutics.

[213] Olsson B., Johansson M., Gabrielsson J., Bolme P. (1988). Pharmacokinetics and Bioavailability of Reduced and Oxidized N-Acetylcysteine. Eur. J. Clin. Pharmacol..

[214] Hara Y., McKeehan N., Dacks P.A., Fillit H.M. (2017). Evaluation of the Neuroprotective Potential of N-Acetylcysteine for Prevention and Treatment of Cognitive Aging and Dementia. J. Prev. Alzheimer’s Dis..

[215] Katz M., Won S.J., Park Y., Orr A., Jones D.P., Swanson R.A., Glass G.A. (2015). Cerebrospinal Fluid Concentrations of N-Acetylcysteine after Oral Administration in Parkinson’s Disease. Park. Relat. Disord..

[216] Dehkordi H.T., Ghasemi S. (2023). Glutathione Therapy in Diseases: Challenges and Potential Solutions for Therapeutic Advancement. Curr. Mol. Med..

[217] Cacciatore I., Baldassarre L., Fornasari E., Mollica A., Pinnen F. (2012). Recent Advances in the Treatment of Neurodegenerative Diseases Based on GSH Delivery Systems. Oxidative Med. Cell. Longev..

[218] Lewerenz J., Maher P. (2011). Control of Redox State and Redox Signaling by Neural Antioxidant Systems. Antioxid. Redox Signal..

[219] Bridges R.J., Natale N.R., Patel S.A. (2012). System X_c_^−^ Cystine/Glutamate Antiporter: An Update on Molecular Pharmacology and Roles within the CNS. Br. J. Pharmacol..

[220] Shi J., He Y., Hewett S.J., Hewett J.A. (2016). Interleukin 1β Regulation of the System X_c_^−^ Substrate-Specific Subunit, XCT, in Primary Mouse Astrocytes Involves the RNA-Binding Protein HuR. J. Biol. Chem..

[221] Dahlmanns M., Dahlmanns J.K., Savaskan N., Steiner H.H., Yakubov E. (2023). Glial Glutamate Transporter-Mediated Plasticity: System X_c_^−^/XCT/SLC7A11 and EAAT1/2 in Brain Diseases. Front. Biosci..

[222] Sprimont L., Janssen P., De Swert K., Van Bulck M., Rooman I., Gilloteaux J., Massie A., Nicaise C. (2021). Cystine–Glutamate Antiporter Deletion Accelerates Motor Recovery and Improves Histological Outcomes Following Spinal Cord Injury in Mice. Sci. Rep..

[223] Stelmashook E.V., Isaev N.K., Genrikhs E.E., Novikova S.V. (2019). Mitochondria-Targeted Antioxidants as Potential Therapy for the Treatment of Traumatic Brain Injury. Antioxidants.

[224] Jou M.J. (2008). Pathophysiological and Pharmacological Implications of Mitochondria-Targeted Reactive Oxygen Species Generation in Astrocytes. Adv. Drug Deliv. Rev..

[225] Hayakawa K. (2022). Commentary: Can Astrocytic Mitochondria Therapy Be Used as Antioxidant Conditioning to Protect Neurons?. Cond. Med..

[226] Zhang H., Chen Y., Li F., Wu C., Cai W., Ye H., Su H., He M., Yang L., Wang X. (2023). Elamipretide Alleviates Pyroptosis in Traumatically Injured Spinal Cord by Inhibiting CPLA2-Induced Lysosomal Membrane Permeabilization. J. Neuroinflamm..

[227] Zhu Y., Wang H., Fang J., Dai W., Zhou J., Wang X., Zhou M. (2018). SS-31 Provides Neuroprotection by Reversing Mitochondrial Dysfunction after Traumatic Brain Injury. Oxidative Med. Cell. Longev..

[228] Zhou J., Wang H., Shen R., Fang J., Yang Y., Dai W., Zhu Y., Zhou M. (2018). Mitochondrial-Targeted Antioxidant MitoQ Provides Neuroprotection and Reduces Neuronal Apoptosis in Experimental Traumatic Brain Injury Possibly via the Nrf2-ARE Pathway. Am. J. Transl. Res..

[229] Cen J., Zhang R., Zhao T., Zhang X., Zhang C., Cui J., Zhao K., Duan S., Guo Y. (2022). A Water-Soluble Quercetin Conjugate with Triple Targeting Exerts Neuron-Protective Effect on Cerebral Ischemia by Mitophagy Activation. Adv. Healthc. Mater..

[230] Hemachandra Reddy P., Manczak M., Kandimalla R. (2017). Mitochondria-Targeted Small Molecule SS31: A Potential Candidate for the Treatment of Alzheimer’s Disease. Hum. Mol. Genet..

[231] Murphy M.P., Smith R.A.J. (2007). Targeting Antioxidants to Mitochondria by Conjugation to Lipophilic Cations. Annu. Rev. Pharmacol. Toxicol..

[232] Tung C., Varzideh F., Farroni E., Mone P., Kansakar U., Jankauskas S.S., Santulli G. (2025). Elamipretide: A Review of Its Structure, Mechanism of Action, and Therapeutic Potential. Int. J. Mol. Sci..

[233] Liu Z.Q., Chan K., Zhou H., Jiang Z.H., Wong Y.F., Xu H.X., Liu L. (2005). The Pharmacokinetics and Tissue Distribution of Sinomenine in Rats and Its Protein Binding Ability In Vitro. Life Sci..

[234] Long L.H., Wu P.F., Chen X.L., Zhang Z., Chen Y., Li Y.Y., Jin Y., Chen J.G., Wang F. (2010). HPLC and LC-MS Analysis of Sinomenine and Its Application in Pharmacokinetic Studies in Rats. Acta Pharmacol. Sin..

[235] Sharma R., Kambhampati S.P., Zhang Z., Sharma A., Chen S., Duh E.I., Kannan S., Tso M.O.M., Kannan R.M. (2020). Dendrimer Mediated Targeted Delivery of Sinomenine for the Treatment of Acute Neuroinflammation in Traumatic Brain Injury. J. Control. Release.

[236] Cassina P., Cassina A., Pehar M., Castellanos R., Gandelman M., De León A., Robinson K.M., Mason R.P., Beckman J.S., Barbeito L. (2008). Mitochondrial Dysfunction in SOD1G93A-Bearing Astrocytes Promotes Motor Neuron Degeneration: Prevention by Mitochondrial-Targeted Antioxidants. J. Neurosci..

[237] Rudnitskaya E.A., Burnyasheva A.O., Kozlova T.A., Peunov D.A., Kolosova N.G., Stefanova N.A. (2022). Changes in Glial Support of the Hippocampus during the Development of an Alzheimer’s Disease-like Pathology and Their Correction by Mitochondria-Targeted Antioxidant SkQ1. Int. J. Mol. Sci..

[238] Muraleva N.A., Stefanova N.A., Kolosova N.G. (2020). SkQ1 Suppresses the P38 MAPK Signaling Pathway Involved in Alzheimer’s Disease-Like Pathology in OXYS Rats. Antioxidants.

[239] Genrikhs E.E., Stelmashook E.V., Alexandrova O.P., Novikova S.V., Voronkov D.N., Glibka Y.A., Skulachev V.P., Isaev N.K. (2019). The Single Intravenous Administration of Mitochondria-Targeted Antioxidant SkQR1 after Traumatic Brain Injury Attenuates Neurological Deficit in Rats. Brain Res. Bull..

[240] Polyzos A., Holt A., Brown C., Cosme C., Wipf P., Gomez-Marin A., Castro M.R., Ayala-Peña S., McMurray C.T. (2016). Mitochondrial Targeting of XJB-5-131 Attenuates or Improves Pathophysiology in HdhQ150 Animals with Well-Developed Disease Phenotypes. Hum. Mol. Genet..

[241] Qin R., Lai X., Xu W., Qin Q., Liang X., Xie M., Chen L. (2025). The Mechanisms and Application Prospects of Astrocyte Reprogramming into Neurons in Central Nervous System Diseases. Curr. Neuropharmacol..

[242] Xia S., Xu C., Liu F., Chen G. (2023). Development of MicroRNA-Based Therapeutics for Central Nervous System Diseases. Eur. J. Pharmacol..

[243] Sun P., Liu D.Z., Jickling G.C., Sharp F.R., Yin K.J. (2018). MicroRNA-Based Therapeutics in Central Nervous System Injuries. J. Cereb. Blood Flow Metab..

[244] Ishii T., Warabi E., Mann G.E. (2019). Circadian Control of BDNF-Mediated Nrf2 Activation in Astrocytes Protects Dopaminergic Neurons from Ferroptosis. Free Radic. Biol. Med..

[245] Saba J., Turati J., Ramírez D., Carniglia L., Durand D., Lasaga M., Caruso C. (2018). Astrocyte Truncated Tropomyosin Receptor Kinase B Mediates Brain-Derived Neurotrophic Factor Anti-Apoptotic Effect Leading to Neuroprotection. J. Neurochem..

[246] Smith J.A., Braga A., Verheyen J., Basilico S., Bandiera S., Alfaro-Cervello C., Peruzzotti-Jametti L., Shu D., Haque F., Guo P. (2018). RNA Nanotherapeutics for the Amelioration of Astroglial Reactivity. Mol. Ther. Nucleic Acids.

[247] Xu T., Chang Y., Wang R., Xu J., Qian D., Yao H., Gan L., Deng S., Lian Q., Ye J. (2025). Lipid Nanoparticle-Mediated Targeted Delivery of MEGF10 SiRNA to Astrocytes Reduced Synaptic Phagocytosis and Promoted Stroke Recovery in Mice. ACS Appl. Mater. Interfaces.

[248] Guo S., Wei F., Sun H., Jin H., Cheng W., Fu C., Wang H., Yin Y. (2025). Astrocyte-Specific Nrf2 Expression Transforms Neurotoxic Reactive Astrocytes to Neuroprotective Phenotype in 3xTg-AD Mice. Glia.

[249] Zhao W., Gasterich N., Clarner T., Voelz C., Behrens V., Beyer C., Fragoulis A., Zendedel A. (2022). Astrocytic Nrf2 Expression Protects Spinal Cord from Oxidative Stress Following Spinal Cord Injury in a Male Mouse Model. J. Neuroinflamm..

[250] Nanou A., Higginbottom A., Valori C.F., Wyles M., Ning K., Shaw P., Azzouz M. (2013). Viral Delivery of Antioxidant Genes as a Therapeutic Strategy in Experimental Models of Amyotrophic Lateral Sclerosis. Mol. Ther..

[251] Xiong W., Garfinkel A.E.M.C., Li Y., Benowitz L.I., Cepko C.L. (2015). NRF2 Promotes Neuronal Survival in Neurodegeneration and Acute Nerve Damage. J. Clin. Investig..

[252] Jiang T., Harder B., Rojo De La Vega M., Wong P.K., Chapman E., Zhang D.D. (2015). P62 Links Autophagy and Nrf2 Signaling. Free Radic. Biol. Med..

[253] Ichimura Y., Komatsu M. (2018). Activation of P62/SQSTM1-Keap1-Nuclear Factor Erythroid 2-Related Factor 2 Pathway in Cancer. Front. Oncol..

[254] Robertson H., Dinkova-Kostova A.T., Hayes J.D. (2020). NRF2 and the Ambiguous Consequences of Its Activation during Initiation and the Subsequent Stages of Tumourigenesis. Cancers.

[255] D’Souza A., Nozohouri S., Bleier B.S., Amiji M.M. (2022). CNS Delivery of Nucleic Acid Therapeutics: Beyond the Blood–Brain Barrier and Towards Specific Cellular Targeting. Pharm. Res..

